# Prognostic and Predictive Value of Integrated Qualitative and Quantitative Magnetic Resonance Imaging Analysis in Glioblastoma

**DOI:** 10.3390/cancers13040722

**Published:** 2021-02-10

**Authors:** Maikel Verduin, Sergey Primakov, Inge Compter, Henry C. Woodruff, Sander M. J. van Kuijk, Bram L. T. Ramaekers, Maarten te Dorsthorst, Elles G. M. Revenich, Mark ter Laan, Sjoert A. H. Pegge, Frederick J. A. Meijer, Jan Beckervordersandforth, Ernst Jan Speel, Benno Kusters, Wendy W. J. de Leng, Monique M. Anten, Martijn P. G. Broen, Linda Ackermans, Olaf E. M. G. Schijns, Onno Teernstra, Koos Hovinga, Marc A. Vooijs, Vivianne C. G. Tjan-Heijnen, Danielle B. P. Eekers, Alida A. Postma, Philippe Lambin, Ann Hoeben

**Affiliations:** 1 Department of Medical Oncology, School for Oncology and Developmental Biology (GROW), Maastricht University Medical Centre+, P.O. Box 5800, 6202 AZ Maastricht, The Netherlands; maikel.verduin@mumc.nl (M.V.); elles.revenich@live.nl (E.G.M.R.); vcg.tjan.heijnen@mumc.nl (V.C.G.T.-H.); ann.hoeben@mumc.nl (A.H.); 2 Department of Radiation Oncology (Maastro), GROW School for Oncology, Maastricht University Medical Centre+, 6202 AZ Maastricht, The Netherlands; inge.compter@maastro.nl (I.C.); marc.vooijs@maastrichtuniversity.nl (M.A.V.); danielle.eekers@maastro.nl (D.B.P.E.); 3 The-D-Lab, Department of Precision Medicine, School for Oncology and Developmental Biology (GROW), Maastricht University, Universiteitssingel 40, 6229 ER Maastricht, The Netherlands; s.primakov@maastrichtuniversity.nl (S.P.); h.woodruff@maastrichtuniversity.nl (H.C.W.); 4 Department of Radiology and Nuclear Medicine, GROW School for Oncology and Developmental Biology, Maastricht University Medical Center+, P.O. Box 5800, 6202 AZ Maastricht, The Netherlands; 5 Department of Clinical Epidemiology and Medical Technology Assessment, Maastricht University Medical Centre+, P.O. Box 5800, 6202 AZ Maastricht, The Netherlands; sander.van.kuijk@mumc.nl (S.M.J.v.K.); bram.ramaekers@mumc.nl (B.L.T.R.); 6 Department of Neurosurgery, Radboud University Medical Center, 6500 HB Nijmegen, The Netherlands; maarten.tedorsthorst@radboudumc.nl (M.t.D.); mark.terlaan@radboudumc.nl (M.t.L.); 7 Department of Medical Imaging, Radboud University Medical Center, 6500 HB Nijmegen, The Netherlands; sjoert.pegge@radboudumc.nl (S.A.H.P.); anton.meijer@radboudumc.nl (F.J.A.M.); 8 Department of Pathology, Maastricht University Medical Center+, P.O. Box 5800, 6202 AZ Maastricht, The Netherlands; jan.beckervordersandforth@mumc.nl (J.B.); ernstjan.speel@mumc.nl (E.J.S.); 9 Department of Pathology, Radboud University Medical Center, 6500 HB Nijmegen, The Netherlands; benno.kusters@radboudumc.nl; 10 Department of Pathology, University Medical Center Utrecht, Utrecht University, 3584CX Utrecht, The Netherlands; w.w.j.deleng@umcutrecht.nl; 11 Department of Neurology, Maastricht University Medical Center+, P.O. Box 5800, 6202 AZ Maastricht, The Netherlands; m.anten@mumc.nl (M.M.A.); martijn.broen@mumc.nl (M.P.G.B.); 12 Department of Neurosurgery, Maastricht University Medical Center+, P.O. Box 5800, 6202 AZ Maastricht, The Netherlands; linda.ackermans@mumc.nl (L.A.); o.schijns@mumc.nl (O.E.M.G.S.); o.teernstra@mumc.nl (O.T.); koos.hovinga@mumc.nl (K.H.); 13 Department of Radiology and Nuclear Medicine, School for Mental Health and Neuroscience, Maastricht University Medical Center+, P.O. Box 5800, 6202 AZ Maastricht, The Netherlands; l.jacobi@mumc.nl

**Keywords:** glioblastoma, radiomics, MRI, prognosis, prediction, machine learning, survival

## Abstract

**Simple Summary:**

Glioblastoma (GBM) is the most malignant primary brain tumor, for which improving patient outcome is limited by a substantial amount of tumor heterogeneity. Magnetic resonance imaging (MRI) in combination with machine learning offers the possibility to collect qualitative and quantitative imaging features which can be used to predict patient prognosis and relevant tumor markers which can aid in selecting the right treatment. This study showed that combining these MRI features with clinical features has the highest prognostic value for GBM patients; this model performed similarly in an independent GBM cohort, showing its reproducibility. The prediction of tumor markers showed promising results in the training set but not could be validated in the independent dataset. This study shows the potential of using MRI to predict prognosis and tumor markers, but further optimization and prospective studies are warranted.

**Abstract:**

Glioblastoma (GBM) is the most malignant primary brain tumor for which no curative treatment options exist. Non-invasive qualitative (Visually Accessible Rembrandt Images (VASARI)) and quantitative (radiomics) imaging features to predict prognosis and clinically relevant markers for GBM patients are needed to guide clinicians. A retrospective analysis of GBM patients in two neuro-oncology centers was conducted. The multimodal Cox-regression model to predict overall survival (OS) was developed using clinical features with VASARI and radiomics features in isocitrate dehydrogenase (*IDH*)-wild type GBM. Predictive models for *IDH*-mutation, 06-methylguanine-DNA-methyltransferase (*MGMT*)-methylation and epidermal growth factor receptor (*EGFR*) amplification using imaging features were developed using machine learning. The performance of the prognostic model improved upon addition of clinical, VASARI and radiomics features, for which the combined model performed best. This could be reproduced after external validation (C-index 0.711 95% CI 0.64–0.78) and used to stratify Kaplan–Meijer curves in two survival groups (*p*-value < 0.001). The predictive models performed significantly in the external validation for *EGFR* amplification (area-under-the-curve (AUC) 0.707, 95% CI 0.582–8.25) and *MGMT*-methylation (AUC 0.667, 95% CI 0.522–0.82) but not for *IDH*-mutation (AUC 0.695, 95% CI 0.436–0.927). The integrated clinical and imaging prognostic model was shown to be robust and of potential clinical relevance. The prediction of molecular markers showed promising results in the training set but could not be validated after external validation in a clinically relevant manner. Overall, these results show the potential of combining clinical features with imaging features for prognostic and predictive models in GBM, but further optimization and larger prospective studies are warranted.

## 1. Introduction

Glioblastoma (GBM) is the most malignant type of primary brain cancer with an incidence of 2–3 cases per 100,000 [1]. Currently, a median survival of fifteen months is achieved with multimodal treatment [2] with a five-year overall relative survival of only 6.8% [3]. However, despite this intensive treatment by neurosurgical intervention, concurrent chemoradiation and adjuvant temozolomide (TMZ) [2], GBM is still considered incurable and recurrence is inevitable. Although major improvements in the treatment of cancer have been made, the current standard-of-care for GBM has largely remained unchanged over the past decade.

GBM is diagnosed using gadolinium contrast-enhanced magnetic resonance imaging (MRI) followed by histopathological examination of tumor tissue specimen obtained after either biopsy or resection. Further characterization of GBM has led to the introduction of the 2016 updated world health organization (WHO) classification of central nervous system tumors [4]. This classification integrates histopathological and morphological examination of the tumor with molecular markers [5]. Thus far, the only predictive marker that has been established into clinical practice is the 06-methylguanine-DNA-methyltransferase (*MGMT*) methylation status, which is predictive of an improved response to alkylating chemotherapy such as TMZ [6]. However, a substantial “grey zone” between *MGMT* methylated and unmethylated patients still exists for which the efficacy of TMZ is still to be determined [7]. Additionally, the presence of a mutation in the isocitrate dehydrogenase (*IDH*) genes—which has been identified as a positive prognostic marker—is linked to dedifferentiated low-grade gliomas which have a distinctly different clinical behavior compared to *IDH* wild-type (WT) GBM [8]. Epidermal growth factor receptor (*EGFR*) amplification is one of the most common genetic alterations (±50%) in GBM [9]. This oncogenic molecular alteration poses a potential therapeutic target but also identifies a biological different subtype of GBM which responds differently to established treatments [10,11]. However, the role of *EGFR* amplification as a prognostic factor still remains controversial [11,12,13] and studies using targeted agents for *EGFR* have so far been unsuccessful but are still ongoing [3]. Additionally, multiple other molecular targets (genetic mutations, amplifications and protein fusion products) have been identified which have either failed in previous clinical trials to improve patient survival or are currently still under investigation [3]. All in all, the integration of molecular markers has led to an improvement in prediction of prognosis and treatment response but a substantial variety remains and no improvement in treatment outcome has been made, which is thought to be due to extensive inter- and intratumor heterogeneity [14]. 

Intratumor heterogeneity complicates treatment efficacy as different regions within the same tumor may contain cells having distinct genetic compositions, transcriptional subtypes and/or proliferation kinetics [3]. Furthermore, temporal heterogeneity has been observed in which changes in the expression of molecular targets occur over time which limits efficacy of targeted approaches [15,16]. In clinical practice and currently used diagnostic techniques and available prognostic models intratumor heterogeneity is not accounted for, since single-cell sequencing is not routinely used. Additionally, it is not clear if molecular GBM heterogeneity can be captured by qualitative and/or quantitative analysis of imaging features.

Imaging techniques have the advantage over standard pathological examination to also analyze the invasive, non-resected, components of GBM and thus capture and analyze the tumor as a whole. Especially temporal heterogeneity of expression of molecular targets cannot be evaluated using routine clinical diagnostics, as re-resection of tumors is not always feasible, making non-invasive imaging an interesting alternative. In order to make a standardized analysis of qualitative MR imaging features, the Visually Accessible Rembrandt Images (VASARI) features were previously developed [17]. VASARI features include tumor size, location and morphology and have previously been shown to be reproducible and of prognostic value [17]. Quantitative imaging analysis using radiomics is an approach to extract imaging features by high-throughput data mining on textures, shapes and intensities [18]. Radiomics has shown prognostic and predictive potential in multiple solid tumors [19,20] including GBM [21]. Furthermore, radiomics features have the potential to analyze the entire tumor and to identify intratumor molecular heterogeneity and underlying biological processes [22,23]. In glioma, radiomics models have been developed to predict tumor grade [24], overall survival (OS) [25] and in GBM trying to predict molecular subtypes [26]. Although *IDH*-mutation status is established as the best prognostic marker in GBM [27], defining different *IDH* wild-type GBM prognostic subgroups is still warranted due to their heterogeneous prognosis and clinical behavior.

The main challenge in developing prognostic and predictive imaging-based models is their generalizability towards all GBM patients treated at different centers. Differences in diagnostic techniques (i.e., scanner vendors and protocols) and treatment and population variety can greatly influence model performances [28]. Due to these challenges, this study utilizes two multi-center datasets to train and validate the developed models.

The objective of this study was to investigate the additive value of qualitative and quantitative imaging heterogeneity analysis to established prognostic clinical features. These data were used to develop a prognostic model for OS in a real-world multi-center GBM population for *IDH1*/2 wild-type (*IDH*-WT) GBM. Furthermore, the value of imaging features as predictor for clinically relevant molecular markers for GBM was explored. 

## 2. Results

### 2.1. Patient and Tumor Characteristics

In total, 142 patients were included in the training cohort and 46 patients in the validation cohort. Median OS was 12.0 months (range, 0–142 months) in the training cohort and 7.3 months (range, 0–30 months) in the validation cohort (log rank *p*-value, 0.001). Patients in the validation cohort more frequently received no adjuvant treatment, but these data were not available for all patients. Patient demographics, received treatment schedules and tumor characteristics are listed in Table 1. Molecular data for a subset of patients are reported as missing due to insufficient formalin-fixed paraffin-embedded (FFPE) material or poor quality or quantity of extracted DNA. VASARI features were available for all patients in both cohorts. For radiomics analysis, T1+Gadolinium and T2- weighted images were available for 105 patients in the training cohort and 44 patients in the validation cohort. MRI characteristics such as types and manufacturers of scanners and imaging protocols are reported in Figures S1 and S2. The numbers of patients that were eligible in the two cohorts for the different models are reported in Table S1.

### 2.2. Prognostic Value of Integrative MRI Imaging Analysis in IDH-Wild Type GBM Population

Median OS was 11.2 months (1.2–132.80 months) in the training cohort and 7.0 months (0.4–29.4 months) in the validation cohort in the *IDH*-WT GBM population. Univariate Cox-regression analysis of VASARI features for OS in the training cohort resulted in 13 features selected for inclusion in multivariable analysis (Table S2). The multivariable Cox-regression model consisted of five VASARI features (Model 1). For radiomics, five radiomics features were selected to predict OS (Model 2) (Table 2). In this study, none of the radiomics features showed evidence of a significant correlation with tumor volume (Figure S3). Additionally, no significant correlation were found between VASARI, radiomics and clinical features (Figure S4). An elaborate explanation of these radiomics features can be found on the Pyradiomics website [29] and in a previous study [30].

Clinical features that were selected in the clinical model were chosen based on previous studies [31] and clinical expertise (Model 3). Next, VASARI features, radiomics features and clinical features multivariable Cox-regression models were combined in different combinations. Model 4 was developed by combining VASARI prognostic index (PI) and Radiomics PI, Model 5 by combining VASARI PI and Clinical PI and Model 6 by combining Radiomics PI and Clinical PI (Model 4–6). Finally, clinical features were combined with the integrated VASARI and radiomics prognostic score to develop an integrated clinical and imaging prognostic model (Model 7) (Table 2). The calibration slope of the PI of Model 7 on the validation set was 0.79 (log-rank test *p*-value 0.27), indicating there is no certainty for the slope in the validation set being different from 1. The joint test of all predictors with the offsetting of the predicted PI results in the *p*-value of 0.23, indicating that there is no evidence of a lack of fit on the validation.

**Figure 1:**
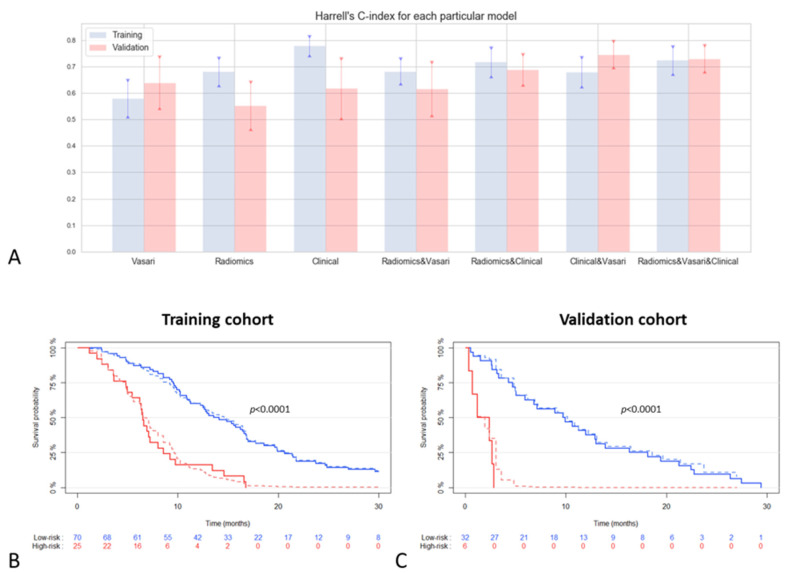
Performance of prognostic models: (**A**) visualization of C-index for all prognostic models (including 95% CI) in the training (*n* = 95) and validation cohort (*n* = 38); (**B**) Kaplan–Meier curve of integrated radiomics, Visually Accessible Rembrandt Images (VASARI) and clinical model (Model 7) in the training cohort and (**C**) validation cohort. Low- and high-risk groups (blue and red lines, respectively) cut-off values were determined by set cut-off (75th percentile) in the training cohort. The solid lines represent the observed survival curves, the dashed the corresponding predicted survival curves.

To assess the reproducibility performance of the prognostic models, all models were tested on the external validation set (*n* = 38) and the discriminative prognostic value in both cohorts was analyzed using Harrell’s C-index (Figure 1A). Model 1 achieved a C-index of 0.61 (95% CI 0.55–0.68) when tested on the whole training cohort (*n* = 129). In order to make a comparison between the different models, the C-index for the VASARI-only model was also calculated using only the patients available in all other models (*n* = 95). In order to visualize the prognostic potential of the integrated imaging and clinical model (Model 7), the data-set was split in a low- and high-risk group at a set cut-off value (75th percentile) of the prognostic index in the training cohort. This same cut-off value was applied to the external validation cohort. Two survival groups could be identified (*p*-value < 0.0001) in both the training and validation cohort (Figure 1B,C).

### 2.3. Predictive Value of Integrative Imaging Analysis

**Figure 2:**
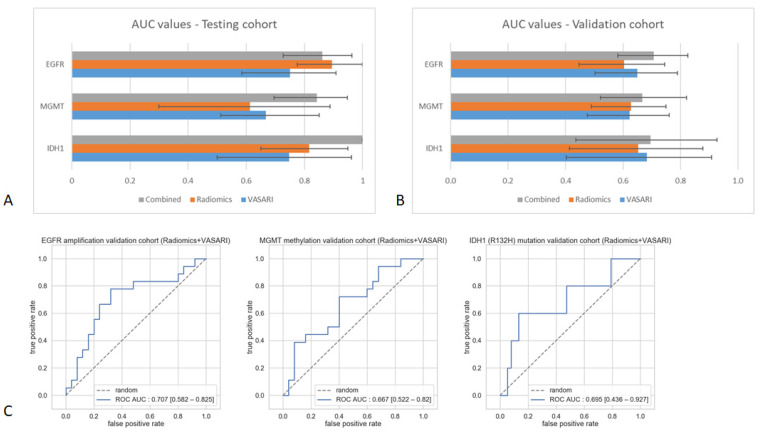
Performance of predictive models: (**A**) Area-under-the-curve (AUC) values and corresponding 95% confidence intervals of different predictive models in the testing cohort and (**B**) in the validation cohort; (**C**) receiver operating characteristic (ROC)-curves of combined VASARI and radiomics model predictive performance in external validation set.

In order to develop the predictive models for molecular markers (*EGFR* amplification, *MGMT*-methylation and *IDH1* mutation), the Maastricht University Medical Center+ (MUMC+) cohort was split into a training (70%) and test (30%) cohort. For the prediction of *EGFR* amplification, in total eleven VASARI features and four radiomics features were selected in the predictive models using the XGBoost machine learning algorithm (Table 3). Both VASARI and radiomics models alone were able to significantly predict *EGFR* amplification in the test dataset (Figure 2A). In the external validation set, both VASARI and radiomics features reached similar results to each other; however, an increased predictive value was observed when both models were combined (area-under-the-curve (AUC) 0.707 (95% CI 0.582–0.825); Figure 2B,C).

The predictive models developed for *MGMT*-methylation status consisted of seven VASARI features (logistic regression analysis) and three radiomics features (XGBoost algorithm) (Table 3). VASARI features alone reached similar predictive values in the test and validation dataset with an AUC of 0.668 (95% CI 0.513–0.850) and 0.622 (95% CI 0.475–0.761) respectively. Radiomics features alone could not predict *MGMT*-methylation in both datasets. An increased predictive value was observed when VASARI features and radiomics features were combined in one predictive model, with an AUC of 0.843 (95% CI 0.696–0.948) in the test dataset but did not perform as well in the external validation dataset (AUC 0.667 (95% CI 0.522–0.820); Figure 2B,C).

For the prediction of the *IDH1* mutation ten VASARI features were included in the multivariate VASARI model and nine radiomics features in the radiomics prediction model developed using the XGBoost machine learning algorithm (Table 3). In the test dataset, only radiomics features reached statistical significance with an ROC AUC of 0.816 (95% CI 0.650–0.950), which improved upon combining with VASARI features (Figure 2A). In the external validation set, neither VASARI nor radiomics features or the combination were able to predict the *IDH1* status (Figure 2B,C). ROC curves for all predictive models in the training and validation cohort are reported in Figures S5 and S6, respectively. 

Next, histogram heterogeneity was assessed to identify whether radiomics features demonstrate significant differences between the outcome groups in a univariate manner. Only for *IDH1* mutation was a significant difference found for two features that could explain the heterogeneity in the outcome. The histograms of heterogeneity for each predictive model and significance values for *IDH1*-mutation are reported in Figures S7 and S8, respectively.

### 2.4. Transparent Reporting of a Multivariable Prediction Model for Individual Prognosis or Diagnosis (TRIPOD) Statement and Radiomics Quality Score

The TRIPOD statement adherences were calculated at 77% for this study. The radiomics quality score (RQS) score calculated for this study was 47%. An overview of point allocation towards the TRIPOD statement and RQS score can be found in Tables S3 and S4, respectively.

## 3. Discussion

Increasing curation rates by optimizing treatment strategies is being hampered by the highly invasive nature and GBM specific inter- and intratumoral molecular heterogeneity. MR imaging is currently the preferred diagnostic imaging technique for GBM. However, integrated standardized qualitative and quantitative analysis of different MR sequences has not yet been introduced into prognostic and predictive GBM models. This study retrospectively analyzed two multi-center GBM patient cohorts to develop integrated clinical and imaging prognostic models and predictive models for clinically relevant molecular markers. 

Combining clinical features with quantitative and qualitative imaging features resulted in the most optimal prognostic model which could be reproduced in the external validation cohort (C-index 0.72 in training cohort and 0.73 in validation cohort). Despite promising results for predicting *EGFR* amplification and *IDH1*-mutation in the test cohort, none of the predictive models for molecular markers were able to predict these markers in a clinically relevant manner in the external validation set.

The prognostic model described in this study is developed for *IDH*-WT GBM patients as this patient group makes up the majority of GBM and exhibits large variation in prognosis and treatment response. This variance is also reflected in statistically significant differences in baseline characteristics for OS and *MGMT*-methylation. However, these differences are also known to exist between centers, in which different treatment decisions and strategies are being implemented. The aim of this study was to investigate the performance of prognostic models in such heterogeneous GBM cohorts. To predict OS, five VASARI features were identified to be of most prognostic relevance. Three of these features are well known prognostic factors and were also previously identified to be negatively associated with OS (involvement of eloquent cortex, multifocality and subependymal extension) and can be attributed to a more invasive growth of the tumor [32,33]. The other selected features, proportion of edema and T1-FLAIR-ratio showed opposite prognostic value in this study when compared to previous studies [33,34,35,36,37]. However, other studies reported no prognostic value for these features and therefore this still remains controversial [32,38].

Radiomics features that were identified to have prognostic value were mainly derived from T2-weighted imaging. This is in line with the hypothesis that the T2-weighted signal corresponds with intratumor heterogeneity and infiltrative tumor growth [39] and this area is accountable for the majority of local recurrences [40]. Therefore, radiomics features from this area are expected to be of importance for survival prediction as was also shown in previous studies [41,42]. The radiomics signature for OS consists of five features, from which two features are the first order Mean (T2-weighted) and Median (T1-weighted) describing the mean and median intensity values after the LLH and HHH wavelet decomposition of the original MR images. The remaining three features quantify gray level zones in an T2-weighted image, more precisely measuring the proportion in the image of the joint distribution of larger size zones with lower gray-level values after image transformation (Laplacian of Gaussian) which is useful for edge detection. These gray level zone features can potentially be associated with the measure of intratumor heterogeneity [43].

In this study, VASARI features alone or radiomics features alone were not able to predict OS in the external validation dataset in a clinically relevant manner. Interestingly, the performance of the prognostic model improved upon combining VASARI, radiomics and clinical features (C-index 0.723 in training cohort and 0.730 in validation cohort) and became clinically relevant. The robustness of this combined model also improved as the model performed similarly in the training- and validation cohort and the uncertainty decreased as represented by a smaller confidence interval of the C-index. Model 5 and 6 report similar performances when compared to the model combining all features. However, the final combined model seems to remain mostly stable between both cohorts, though the actual additive value should be further validated in larger patient cohorts. The combined model was also able to accurately split the two cohorts in a high- and low-risk group (*p*-value < 0.001) (Figure 1B,C). Previous studies also observed that combining clinical features with imaging features improves the prognostic value of the model [42,44,45,46,47]. The model developed in this study performed similar or better compared to previous findings, even after external validation in a heterogeneous patient cohort. This highlights the clinical relevant potential of combining these features into a multimodal prognostic model which can potentially be applied in clinical practice.

As a proof-of-concept study, this study investigated the capability of VASARI and radiomics features to link phenotype to genotype and predict clinically relevant molecular markers, *IDH1*-mutation, *MGMT*-methylation and *EGFR* amplification, by machine learning approaches. Overall, the predictive models had promising performance on the test set, especially when VASARI and radiomics features were combined (Figure 2A). Unfortunately, none of the developed models were able to predict in the external validation set in a clinically relevant manner with a wide spread in confidence intervals of the AUC values (Figure 2B,C). In order for a model predicting molecular markers to be clinically relevant, much higher AUC values are desired. Since the presence of the molecular markers has biological consequences on tumor growth and development, specific imaging techniques that reflect biological processes have shown more promising results in the prediction of these markers and should therefore be used for further research. Perfusion-weighted and/or diffusion-weighted MRI features have been used to predict *EGFR* amplification [48,49,50] and *MGMT*-methylation [51], whereas MR spectroscopy [52] and amino acid tracer PET imaging (FET–PET) [53] can predict *IDH1* mutation status due to its effects on tumor metabolism.

In addition, by analyzing the heterogeneity histogram for *EGFR* amplification based on the validation cohort, we can notice that none of the radiomics features has demonstrated significant difference between the outcome groups in the univariate manner. Heterogeneity histogram for *MGMT*-methylation also did not demonstrate the significant difference between the outcome groups. For *IDH1* mutation, however, we can point out a significant difference (*p* < 0.05) for T2_original_firstorder_10Percentile, T1_wavelet_HLL_glcm_DifferenceAverage features, which indicates the ability of these features to reflect the heterogeneity in the outcome (Figure S8). These findings also highlight the value of multivariate predictive analysis.

The overall RQS of 47% achieved in this study is higher than generally reported in neuro-oncology radiomics studies [54].

The main strength of this study includes the usage of two independent multicenter datasets. Though the performance of previous prognostic models based on VASARI or radiomics features is generally better, most of these studies only use internal validation methods and lack validation in an independent external dataset [34,55]. The same applies to the performance of predictive models for molecular markers. However, the fact that the promising results for the predictive models in this study in the testing cohort could not be replicated in the external validation cohort stresses the importance of external validation. Additionally, most studies use a more homogeneous patient cohort, for example, with regards to treatment characteristics, whereas this present study comprises two heterogeneous cohorts which more reflects daily clinical practice. For example, corticosteroid usage is known to decrease the amount of edema, therefore altering the T2-weighted signal, which can influence both VASARI and radiomics features. Previous studies either do not mention corticosteroid usage or exclude patients using corticosteroids [36,37] even though a significant amount of GBM patients are known to use corticosteroids. Furthermore, multiple studies only use single-institute data in which real-life heterogeneity between MRI acquisition is not represented [56] which is important for the generalizability of radiomics models.

Several limitations should be taken into account when considering the results of this study. The main limitation of this study is the number of patients that were included. Though for the OS models the number of patients is in accordance with the majority of previous studies, especially the limited available molecular data in the external validation set limits the validation capacity of the predictive models. Especially *IDH1*/2 mutations rarely occur in both cohorts, which is to be expected in GBM, leading to wide confidence intervals and complications in the validation of the model. Future studies using a larger *IDH*-mutated cohort are needed to accurately test the models developed in this study. Next, the fact that this study is a retrospective study poses a potential selection bias. Additionally, the Karnofsky Performance Score (KPS) is an established prognostic feature which could not be included in this study due to lack of reporting of the KPS in patient files during the time period used for this study. Furthermore, it could be stated that a limitation of this study was the lack of advanced MRI sequences such as diffusion- and perfusion-weighted imaging and PET-MRI. However, this study specifically chose to focus on the relevance of conventional MRI images as these are widely available in clinical centers. Furthermore, MRI radiomics features are known to be dependent on differences in MRI scanners and scanning protocols. The images used in this study were collected from more than ten different hospitals over a ten-year time-period resulting in large differences in technical MRI characteristics. Again, even though this limits the performance of radiomics, an ideal prognostic and predictive model should not be dependent on homogeneous data. These differences in MRI acquisition methods are present in the real-life multicenter setting and should be accounted for in order to provide a relevant, clinical applicable model.

In order to further improve the prognostic and predictive potential of non-invasive imaging models, several steps need to be taken. First of all, larger (big data) datasets and preferably prospective studies are warranted to develop more accurate and generalizable models. This could pose a challenge, especially in less common types of cancer such as GBM. Next, the first studies on radiomics have been conducted on computed tomography (CT) imaging, which can be quantified using standardized Hounsfield units. For MRI radiomics, such a unit does not exist which poses problems due to inter- and intra-scanner variability. Multiple pre-processing methods have been developed, though not all radiomics features were shown to be robust between different pre-processing approaches [57,58,59]. This calls for a generalized pre-processing pipeline and focus on features that are shown to be robust. Robust features and normalization methods can be achieved by applying phantom studies to account for differences between MRI acquisition protocols [60]. Tumor delineation poses another important aspect of radiomics feature extraction. Manual delineation is still generally seen as the golden standard, though a substantial inter-observer variability exists, despite international guidelines on tumor delineation [61] and it is a time consuming process. It has been shown that this inter-observer variation influences the radiomics analysis in multiple tumors [62]. Automatic segmentation methods using a deep learning neural network approach are widely developed and can be beneficial in future radiomics studies and its clinical applicability by decreasing workload on clinicians and inter-observer variability [63,64]. This is expected to lead to more robust radiomics features due to standardization of the delineation method.

Parallel to the establishment of MR signatures that are able to predict clinically significant expression of specific biomarkers, there is a need for imaging signatures that capture the level of intratumoral heterogeneity. However, it needs to be emphasized that is not yet clarified how to quantify GBM MR imaging heterogeneity and moreover how to non-invasively analyze the level of intratumoral heterogeneous expression of predictive markers, since the golden standard, single cell RNA sequencing, is missing in standard of care. By extracting radiomics features from the whole tumor and the surrounding area of edema we identified several features that are associated with intratumor heterogeneity. However, different steps could be taken to include more aspects of tumor heterogeneity. Improved performance of radiomics has been reported when features are extracted from distinct tumor areas (active tumor, necrosis and edema) separately [65,66], though this is a more labor-intensive approach which might limit its clinical applicability. In this aspect, automatic segmentation algorithms have shown to be useful for prognostic radiomics modelling [47]. Additionally, more biologically relevant MRI sequences such as diffusion- or perfusion-weighted MRI have been shown to outperform radiomics models based on conventional MRI [25]. These approaches should be taken into account in future studies as they will be able to encompass more features concerning intratumor heterogeneity [67] and have shown improved performance with regards to predicting prognosis and molecular markers. Ultimately, studies correlation pathological and genetic examination of multiregional biopsies towards imaging features are needed to study the value of imaging features for tumor heterogeneity.

## 4. Materials and Methods

### 4.1. Patient Population

All patients treated by the neuro-oncology team of the Maastricht University Medical Centre (Maastricht UMC+, Maastricht, the Netherlands) between January 2004 and August 2014 for a glioblastoma (WHO grade IV) were considered for inclusion in the retrospective training cohort. Patients were excluded if no diagnostic, pre-operative MRI-images were available (minimum T1+Gadolinium and T2-weighed imaging), if survival data were unknown or no histological diagnosis was available. All patient records were reviewed considering patient and tumor characteristics, received treatments and survival data. The external validation cohort was constructed using the same criteria on an independent dataset of patients treated in Radboud University Medical Center (Radboudumc, Nijmegen, The Netherlands) in the same time period. Both Maastricht UMC+ and Radboudumc are academic reference centers for GBM patients in the Netherlands, implying MRI-images were also obtained in hospitals that refer their patients to these academic centers. Numbers of patients used for each analysis are reported in Table S1. The requirement for informed consent for this retrospective study was waived by the medical ethics committee of the MUMC+ (METC 16-4-022).

### 4.2. Image Acquisition and Qualitative Imaging Feature Assessment

**Figure 3:**
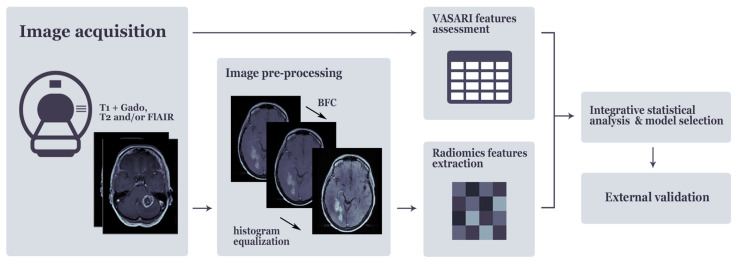
Quantitative and qualitative imaging analysis pipeline.

Pre-operative MRI images were collected, pseudonymized and pooled in a database combining MRI images from different types and manufacturers of scanners using different imaging protocols to reflect the real-life inter-center heterogeneity (Figures S1 and S2). A quantitative and qualitative imaging analysis pipeline was set-up (Figure 3). All diagnostic MRI-scans were analyzed by dedicated neuro-radiologists (SP, AJ, AP), blinded for outcome and scored using the VASARI Imaging Features. A previous study conducted by the VASARI research project group showed a strong overall inter-observer agreement among six readers for the VASARI features [29]. When needed, multi-categorical and continuous VASARI features were recoded into different groups based on their clinical relevance prior to the start of analysis (Table S5).

### 4.3. Tumor Delineation, Image Pre-Processing and Extraction of Radiomics Features

Using Osirix Lite (Pixmeo SARL, Bernex, Switzerland) and MiM software (version 7.0.4, MIM Software Inc., Cleveland, OH, USA), regions of interests (enhancing tumor on T1+Gadolinium images and combined tumor/edema portion on T2-weighted images) were manually delineated on all diagnostic MRI-images of the training and validation cohort, supervised by two experienced neuro-radiation oncologists (DE, IC).

Using Python 3.7 and the dedicated packages (cv2 version 4.1.0, https://pypi.org/project/opencv-python/, (accessed on 23 December 2020)), SimpleITK version 1.2.0 (https://simpleitk.org/, (accessed on 23 December 2020)) and scikit-image version 0.14.2, (https://scikit-image.org/, (accessed on 23 December 2020)), an image pre-processing routine was developed to handle the broad variability of image acquisition and reconstruction parameters. 

At first, spatial resolution of the images was normalized with respect to the image sequence (final pixels are: 0.449 mm^2^ and slice thickness of: 5.5 mm). The mode of the pixel spacing and slice thickness distributions from the Maastricht UMC+ cohort were used as reference values for the resampling procedure to minimize the number of resampled images. A bicubic interpolation over 4 × 4 pixel neighborhood was used for both upsampling and downsampling. In order to correct the low frequency intensity non-uniformity, which is intrinsic for MRI images, the N4 bias field correction algorithm was used [68]. Furthermore, the histogram equalization method implemented in the scikit-image 0.15.0 package [69] was used to enhance the contrast of MRI images [70]. As the last step of the pre-processing routine, image intensities were normalized using Z-score standardization method [71]. A pre-processing routine was applied to both cohorts, where parameters (mu, sigma) for the Z-score transformation were evaluated on the training cohort and transferred to the validation cohort. Parameters used are T1 mu = 0.1904, T1 sigma = 0.2313, T2 mu = 0.2009 and T2 sigma = 0.2448.

In order to obtain the quantitative imaging features, an open-source Pyradiomics 2.2.0 python package for the radiomics features extraction was utilized [72]. Using the dedicated MRI settings, features from following feature classes were extracted: First Order Statistics, Shape-based (2D and 3D), Gray Level Cooccurence Matrix (GLCM), Gray Level Run Length Matrix (GLRLM), Gray Level Size Zone Matrix (GLSZM), Gray Level Dependence Matrix (GLDM), Neighboring Gray Tone Difference Matrix (NGTDM). Along with the original features Laplacian of Gaussian (LoG) (sigma: (2.0,3.0,4.0,5.0) and Wavelet filters were activated resulting in a total of 1197 features per patient. A detailed mathematical feature description as provided by Aerts et al. 2014 [30].

### 4.4. Molecular Markers

Archival formalin-fixed paraffin-embedded (FFPE) tissue samples were analyzed for tumor percentage by an experienced neuro-pathologist (JB). DNA was extracted from FFPE tissue using the Cobas method (Roche, Bazel, Switzerland) and DNA concentration was quantified using Qubit Fluorometer (Life Technologies, Waltham, MA, USA). Next-generation sequencing (NGS) was performed using the Ion AmpliSeq Cancer Hotspot Panel v2 (Life Technologies) as previously described [73]. For the purpose of this study, the data were analyzed for the presence of an *IDH1* (R132H) mutation (minimum coverage 500×) which was manually checked using Integrative Genomics Viewer (IGV). *EGFR* amplification was assessed using SNPitty, an open-source web application for interactive B-allele frequency and copy number visualization of NGS data, by comparing the number of reads in the *EGFR* locus to the surrounding regions [74]. *MGMT* methylation status was assessed using methylation-specific multiplex ligation-dependent probe amplification (MS-MLPA) as previously described [75]. In case NGS data was not available for a sample, MLPA was also used to assess *IDH1* mutation status and *EGFR* amplification.

### 4.5. Statistical Analysis

Statistical analysis for differences between baseline characteristics was performed using double-sided T-test for “age at diagnosis”. Fisher’s exact test was used for all other binary variables (sex, type of surgery, adjuvant treatment and molecular markers).

Overall survival (OS) was defined as the time between the initial surgical intervention after diagnosis and the date of death (confirmed by the Municipal Personal Records Database). Patients that survived were censored at the moment of the last follow-up measurement. To develop a prognostic model, analysis was focused on the *IDH*-WT GBM samples.

OS analysis was performed using R (version 4.0.2., R Studio, Boston, MA, USA), employing the packages stats, survival, survminer, rms, pec and survcomp. VASARI features were tested in univariate Cox-regression analysis to determine the hazard ratio (HR) of each feature individually on the training cohort. Each feature with a *p*-value of ≤ 0.2 was considered for inclusion in the multivariable analysis. Resulting VASARI features were used for multivariable Cox-regression analysis with fast backward elimination (removal alpha < 0.2) on the training set. Radiomics features from T1- and T2-weighted images were combined and normalized with the Z-score transformation, where coefficients evaluated on the train set were transferred to the validation set. Highly correlated features exceeding the Spearman’s rank correlation of rs = 0.85 were eliminated. Resulting radiomics features, were used for multivariable Cox-regression analysis with fast backward elimination for the training set [76] (Model 1–3) All clinical features were entered into the Cox-regression model to develop the Clinical model on the training set.. A prognostic Index (PI) for all models developed on the training set was calculated for training and validation datasets, where the PI was defined as $\underset{i}{\sum }\beta_{i}x_{i}$ for each individual model. For the combined models, the PI of the individual models was used as a feature along with the PI for the individual model it was combined with in Cox-regression analysis [77]. Similarly, for a combined clinical/VASARI/radiomics model (Model 7), VASARI PI was used as a feature along the radiomics PI and clinical PI. Next, the models were validated using multiple-step approach [78]. Calibration slope was assessed using the Log-rank (LR) test. Model’s misspecification was evaluated by performing the Cox regression on the individual features of the signature in the validation dataset with offsetting the validation PI [78].

Overall model performance for discriminating survival groups was evaluated with Harrell’s C-index. To display the potential discrimination between survival groups Kaplan–Meier (KM) curves were used with the threshold value based on 75th percentile of training PI’s in order to identify a high-risk group using our model. Significance of the split was estimated using the LR test. In addition, predicted survival curves for each risk group were plotted. The PI is used to estimate the survival curve, which is then averaged over the entire risk-group. These curves are plotted alongside the observed KM-curves. The correlation between radiomics features and tumor volume was assessed using Spearman’s rank correlation. This was investigated since previous studies have shown some radiomics features to be surrogate markers for tumor volume and not independent prognostic features [79]. Correlation between VASARI features, radiomics features and clinical features were assessed using the point-biserial correlation coefficient.

Python 3.7 was used to develop and validate the predictive models. Patients with unknown outcomes (molecular markers) were excluded from the analysis. At first, highly correlated features (rs > 0.85) were eliminated, in which the feature with the lower AUC value in univariate ROC analysis was removed and resulting features were normalized using Z score on the MUMC+ cohort. Shift/scale parameters of individual features are available upon request. As the second step, the MUMC+ cohort was split randomly into train and test sets with a 70/30 ratio and label stratification. In the third step, to obtain the feature importance scores, a random forest model with the random-sampled initialization of hyper parameters (each iteration parameter was randomly sampled from the hyper parameter ranges: number of estimators (20,300), max depth (2,6)) was fitted 1000 times resulting in the cumulative feature importance histogram. Based on the feature importance rank, the 20 most important features were selected for the further evaluation. In order to find the best performing model in the fourth step, Xgboost, Random Forest and Logistic regression algorithms were initialized with the random-sampling of hyper parameters (Table S6), trained and tested 1000 times. In order to overcome a “lucky split bias”, step 2 (the random splitting of the cohorts) followed by model testing was repeated 10 times for the top 5 performing models from step 4, representing the cross validation technique.

Combined model was achieved by ensembling VASARI and radiomics models using averaging of VASARI and radiomics predicted probabilities. To evaluate performance of the predictive models, the area under the receiver operating characteristic (ROC) curve, or AUC, was calculated. Bootstrapping technique with 100 iterations was utilized to estimate ROC AUC 95% confidence intervals on test and validation datasets.

Additionally, to visualize the ability of radiomics features of capturing the outcome heterogeneity in a univariate manner and contribute to concept of explainable radiomics, we visualized the outcome heterogeneity through selected radiomics features by plotting the distribution of feature values for each particular feature of each binary outcome. The significance of the difference in the mean values was evaluated by performing the Mann–Whitney test with Bonferroni correction.

### 4.6. TRIPOD Statement and Radiomics Quality Score (RQS)

To assess the quality of the conducted study, a radiomics quality score (RQS) was calculated. The RQS is a checklist consisting of 16 components to assess the validity of the radiomics workflow and (external) validation of the models [19,80]. Furthermore, the checklist recommended in transparent reporting of a multivariable prediction model for individual prognosis or diagnosis (TRIPOD) was assessed [81].

## 5. Conclusions

In the present study, the potential of non-invasive quantitative and qualitative imaging features to predict prognosis and clinically relevant molecular markers was investigated in a real-life heterogeneous GBM patient cohort. The integrated prognostic model, including clinical and imaging features, showed the most promising performance which was reproducible and most robust between both datasets. However, further improvements and larger prospective studies are needed before this model can be used in daily clinical practice. Using imaging features to predict molecular markers showed promising results in the testing set but could not be validated on the external validation set and warrants additional validation in larger GBM cohorts.

## Figures and Tables

**Table 1:** Overview of patient, treatment and tumor characteristics in the training and validation cohort.

Demographics	Training Cohort (*n* = 142)	Validation Cohort (*n* = 46)	*p*-Value
Median age at diagnosis (range)	61.4 years (15–85)	61.7 years (18–81)	0.991
Sex (%)	Male: 85 (59.9%) Female: 57 (40.1%)	Male: 29 (63.0%) Female: 17 (37.0%)	0.258
**Treatment characteristics**			
Surgical treatment (%)	Biopsy: 54 (38.0%) Debulking: 88 (62.0%)	Biopsy: 17 (37.0%) Debulking: 29 (63.0%)	0.112
Adjuvant treatment (%)	STUPP completed: 67 (47.2%) STUPP not completed or Non-STUPP regimen: 75 (52.8%)	STUPP completed: 17 (37.0%) STUPP not completed or Non-STUPP regimen: 16 (34.8%) Missing: 13 (28.2%)	0.288
**Tumor characteristics**			
*Isocitrate dehydrogenase (IDH1)* (R132H) mutation status (%)	*IDH1/2*-WT: 129 (91.5%) *IDH1*-mutation: 12 (8.5%) Missing: 1	*IDH1/2*-WT: 39 (84.8%) *IDH1*-mutation: 5 (10.9%) Missing: 2	1.000
*Methylguanine methyltransferase (MGMT)*-methylation status (%)	*MGMT*-methylated: 37 (26.2%) *MGMT* non-methylated: 104 (73.8%) Missing: 1	*MGMT*-methylated: 18 (39.1%) *MGMT* non-methylated: 26 (56.5%) Missing: 2	0.045
*Epidermal growth factor receptor (EGFR)* amplification status (%)	*EGFR* amplified: 47 (37.3%) *EGFR* non-amplified: 79 (62.7%) Missing: 16	*EGFR* amplified: 20 (43.5%) *EGFR* non-amplified: 26 (56.5%) Missing: 0	0.738

**Table 2:** Multivariate Cox-regression model using Visually Accessible Rembrandt Images (VASARI), radiomics and/or clinical features for overall survival (OS) prediction in isocitrate dehydrogenase wild type (*IDH*-WT) glioblastoma (GBM) patients in different prognostic models based on the training cohort (*n* = numbers of patients used for model development).

Prognostic Model Variables	Hazard Ratio (95% CI)	*p*-Value
Model 1: VASARI features model (*n* = 129)		
Involvement of eloquent cortex	1.28 (0.88–1.87)	0.198
Multifocality	1.72 (0.97–3.05)	0.064
Subependymal extension	1.75 (1.21–2.53)	0.003
Low proportion of edema	Reference	Reference
Medium proportion of edema	1.09 (0.75–1.61)	0.653
High proportion of edema	0.45 (0.24–0.83)	0.011
Increased T1FLAIR-ratio	0.59 (0.37–0.94)	0.026
Model 2: Radiomics features model (*n* = 95)		
T1_wavelet.HHH_firstorder_Median	1.04 (0.81–1.3)	0.754
T2_log.sigma.2.0.mm.3D_glszm_LargeAreaLowGrayLevelEmphasis	1.00 (0.83–1.2)	0.958
T2_log.sigma.3.0.mm.3D_glszm_LargeAreaLowGrayLevelEmphasis	1.33 (1.07–1.6)	0.009
T2_wavelet.LLH_firstorder_Mean	1.70 (1.32–2.2)	0.001
T2_wavelet.HHL_glszm_LargeAreaLowGrayLevelEmphasis	0.92 (0.75–1.1)	0.404
Model 3: Clinical features model (*n* = 95)		
Sex (male vs. female)	1.12 (0.70–1.77)	0.644
Type of surgery (resection vs. biopsy)	0.48 (0.31–0.76))	0.002
Age at diagnosis (>70 vs. <70)	1.10 (0.60–2.02)	0.749
Adjuvant treatment (non-STUPP vs. STUPP)	4.92 (2.79–8.67)	0.001
Methylguanine methyltransferase (*MGMT*)-methylation	0.61 (0.35–1.06)	0.082
Model 4: Integrated imaging model (VASARI + radiomics) (*n* = 95)		
VASARI prognostic score	2.2 (1.4–3.4)	<0.001
Radiomics prognostic score	2.92 (1.9–4.5)	<0.001
Model 5: Integrated VASARI and clinical model (*n* = 95)		
VASARI prognostic score	2.0 (1.3–3.2)	0.003
Clinical prognostic score	2.7 (1.8–3.9)	<0.001
Model 6: Integrated Radiomics and clinical model (*n* = 95)		
Radiomics prognostic score	2.6 (1.7–4.0)	<0.001
Clinical prognostic score	2.8 (1.9–4.1)	<0.001
Model 7: Integrated imaging and clinical model (*n* = 95)		
VASARI prognostic score	2.1 (1.4–3.3)	<0.001
Radiomics prognostic score	3.0 (1.9–4.7)	<0.001
Clinical prognostic score	2.1 (1.4–3.3)	<0.001

**Table 3:** Selected VASARI and radiomics features in predictive models for epidermal growth factor receptor (*EGFR*) amplification, methylguanine methyltransferase(*MGMT)*-methylation and isocitrate dehydrogenase 1(*IDH1)* mutation in GBM patients in the training cohort.

VASARI Features	Radiomics Features
*EGFR* amplification (*n =* 64)	
Size: Major Axis, Minor Axis, Mean Minor Axis, Median Major Axis Location: Eloquent location, Midline cross of enhancing tumor Morphology: Proportion necrosis Tumor characteristics: Hemorrhage, Subependymal Extension, Pial invasion, Definition enhancing margin	T1+Gado: wavelet-HLH_glcm_Correlation T2: log-sigma-2-0-mm-3D_gldm_LargeDependenceLowGrayLevelEmphasis; wavelet-LLH_glcm_ClusterShade; wavelet-LLH_firstorder_Skewness
*MGMT-methylation* (*n* = 74)	
Size: Major Axis, Minor Axis, Median Major Axis, Mean Major Axis Morphology: Proportion non-enhancing tumor Tumor characteristics: Deep white matter invasion, Subependymal extension	T1+Gado: no features selected T2: wavelet-HLL_gldm_LargeDependenceHighGrayLevelEmphasis; log-sigma-5-0-mm-3D_glrlm_HighGrayLevelRunEmphasis; log-sigma-5-0-mm-3D_glszm_SmallAreaHighGrayLevelEmphasis
*IDH1* mutation (*n* = 72)	
Size: Minor Axis, Major Axis Location: Tumor side, Eloquent location Morphology: Proportion non-enhancing tumor, Proportion Edema Tumor characteristics: Pial invasion, Thickness Enhancing Margin, Definition enhancing margin, T1-FLAIR-ratio	T1+Gado: wavelet-HLL_glcm_Contrast; wavelet-HLL_glcm_DifferenceAverage T2: log-sigma-2-0-mm-3D_firstorder_90Percentile; Original_glrlm_LongRunHighGrayLevelEmphasis; log-sigma-3-0-mm-3D_firstorder_90Percentile; original_firstorder_10Percentile; log-sigma-4-0-mm-3D_firstorder_Uniformity; wavelet-HLL_gldm_DependenceEntropy; wavelet-HLL_glcm_Correlation

## Data Availability

The data presented in this study are available on request from the corresponding author. The data are not publicly available due to privacy restrictions.

## Supplementary Materials

The following are available online at https://www.mdpi.com/2072-6694/13/4/722/s1, Figure S1: Imaging heterogeneity in MUMC+ cohort, Figure S2: Imaging heterogeneity in Radboudumc cohort, Figure S3: Correlation matrix between radiomics features and tumor volume, Figure S4: Correlation matrix between VASARI features, clinical features and radiomics features. Figure S5: ROC curves for all predictive models in the test dataset, Figure S6: ROC curves for all predictive models in the validation dataset, Figure S7: Heterogeneity histograms from selected radiomics features in predictive models, Figure S8: Mann–Whitney test for significance of histogram heterogeneity of radiomics features in predicting IDH1 mutation status, Table S1: Number of patients included for development and validation of each model, Table S2: Univariate Cox-regression analysis of VASARI features for OS in IDH-WT GBM population, Table S3: TRIPOD statement, Table S4: Radiomics Quality Score (RQS), Table S5: Definitions of VASARI features as used in this study, Table S6: Tuned hyperparameters for predictive models.

## References

[1] Urbanska K., Sokolowska J., Szmidt M., Sysa P. (2014). Glioblastoma multiforme—An overview. Contemp. Oncol. (Pozn).

[2] Stupp R., Mason W.P., van den Bent M.J., Weller M., Fisher B., Taphoorn M.J., Belanger K., Brandes A.A., Marosi C., Bogdahn U. (2005). Radiotherapy plus concomitant and adjuvant temozolomide for glioblastoma. N. Engl. J. Med..

[3] Wen P.Y., Weller M., Lee E.Q., Alexander B.M., Barnholtz-Sloan J.S., Barthel F.P., Batchelor T.T., Bindra R.S., Chang S.M., Chiocca E.A. (2020). Glioblastoma in adults: A Society for Neuro-Oncology (SNO) and European Society of Neuro-Oncology (EANO) consensus review on current management and future directions. Neuro Oncol..

[4] Louis D.N., Perry A., Reifenberger G., von Deimling A., Figarella-Branger D., Cavenee W.K., Ohgaki H., Wiestler O.D., Kleihues P., Ellison D.W. (2016). The 2016 World Health Organization Classification of Tumors of the Central Nervous System: A summary. Acta Neuropathol..

[5] Weller M., van den Bent M., Preusser M., Le Rhun E., Tonn J.C., Minniti G., Bendszus M., Balana C., Chinot O., Dirven L. (2020). EANO guidelines on the diagnosis and treatment of diffuse gliomas of adulthood. Nat. Rev. Clin. Oncol..

[6] Hegi M.E., Diserens A.C., Gorlia T., Hamou M.F., de Tribolet N., Weller M., Kros J.M., Hainfellner J.A., Mason W., Mariani L. (2005). MGMT gene silencing and benefit from temozolomide in glioblastoma. N. Engl. J. Med..

[7] Hegi M.E., Genbrugge E., Gorlia T., Stupp R., Gilbert M.R., Chinot O.L., Nabors L.B., Jones G., Van Criekinge W., Straub J. (2019). MGMT Promoter Methylation Cutoff with Safety Margin for Selecting Glioblastoma Patients into Trials Omitting Temozolomide: A Pooled Analysis of Four Clinical Trials. Clin. Cancer Res..

[8] Dahlrot R.H., Kristensen B.W., Hjelmborg J., Herrstedt J., Hansen S. (2013). A population-based study of high-grade gliomas and mutated isocitrate dehydrogenase 1. Int. J. Clin. Exp. Pathol..

[9] Lassman A.B., Aldape K.D., Ansell P.J., Bain E., Curran W.J., Eoli M., French P.J., Kinoshita M., Looman J., Mehta M. (2019). Epidermal growth factor receptor (EGFR) amplification rates observed in screening patients for randomized trials in glioblastoma. J. Neuro Oncol..

[10] Eskilsson E., Rosland G.V., Solecki G., Wang Q., Harter P.N., Graziani G., Verhaak R.G.W., Winkler F., Bjerkvig R., Miletic H. (2018). EGFR heterogeneity and implications for therapeutic intervention in glioblastoma. Neuro Oncol..

[11] Saadeh F.S., Mahfouz R., Assi H.I. (2018). EGFR as a clinical marker in glioblastomas and other gliomas. Int. J. Biol. Mark..

[12] Armocida D., Pesce A., Frati A., Santoro A., Salvati M. (2020). EGFR amplification is a real independent prognostic impact factor between young adults and adults over 45yo with wild-type glioblastoma?. J. Neuro Oncol..

[13] Hoffman D.I., Abdullah K.G., McCoskey M., Binder Z.A., O’Rourke D.M., Desai A.S., Nasrallah M.P., Bigdeli A., Morrissette J.J.D., Brem S. (2019). Negative prognostic impact of epidermal growth factor receptor copy number gain in young adults with isocitrate dehydrogenase wild-type glioblastoma. J. Neuro Oncol..

[14] Patel A.P., Tirosh I., Trombetta J.J., Shalek A.K., Gillespie S.M., Wakimoto H., Cahill D.P., Nahed B.V., Curry W.T., Martuza R.L. (2014). Single-cell RNA-seq highlights intratumoral heterogeneity in primary glioblastoma. Science.

[15] Qazi M.A., Vora P., Venugopal C., Sidhu S.S., Moffat J., Swanton C., Singh S.K. (2017). Intratumoral heterogeneity: Pathways to treatment resistance and relapse in human glioblastoma. Ann. Oncol..

[16] Draaisma K., Chatzipli A., Taphoorn M., Kerkhof M., Weyerbrock A., Sanson M., Hoeben A., Lukacova S., Lombardi G., Leenstra S. (2020). Molecular Evolution of IDH Wild-Type Glioblastomas Treated With Standard of Care Affects Survival and Design of Precision Medicine Trials: A Report From the EORTC 1542 Study. J. Clin. Oncol..

[17] Wangaryattawanich P., Hatami M., Wang J., Thomas G., Flanders A., Kirby J., Wintermark M., Huang E.S., Bakhtiari A.S., Luedi M.M. (2015). Multicenter imaging outcomes study of The Cancer Genome Atlas glioblastoma patient cohort: Imaging predictors of overall and progression-free survival. Neuro Oncol..

[18] Aerts H.J. (2016). The Potential of Radiomic-Based Phenotyping in Precision Medicine: A Review. JAMA Oncol..

[19] Lambin P., Leijenaar R.T.H., Deist T.M., Peerlings J., de Jong E.E.C., van Timmeren J., Sanduleanu S., Larue R., Even A.J.G., Jochems A. (2017). Radiomics: The bridge between medical imaging and personalized medicine. Nat. Rev. Clin. Oncol..

[20] Rogers W., Thulasi Seetha S., Refaee T.A.G., Lieverse R.I.Y., Granzier R.W.Y., Ibrahim A., Keek S.A., Sanduleanu S., Primakov S.P., Beuque M.P.L. (2020). Radiomics: From qualitative to quantitative imaging. Br. J. Radiol..

[21] Chaddad A., Kucharczyk M.J., Daniel P., Sabri S., Jean-Claude B.J., Niazi T., Abdulkarim B. (2019). Radiomics in Glioblastoma: Current Status and Challenges Facing Clinical Implementation. Front. Oncol..

[22] Peeken J.C., Molina-Romero M., Diehl C., Menze B.H., Straube C., Meyer B., Zimmer C., Wiestler B., Combs S.E. (2019). Deep learning derived tumor infiltration maps for personalized target definition in Glioblastoma radiotherapy. Radiother. Oncol..

[23] Grossmann P., Stringfield O., El-Hachem N., Bui M.M., Rios Velazquez E., Parmar C., Leijenaar R.T., Haibe-Kains B., Lambin P., Gillies R.J. (2017). Defining the biological basis of radiomic phenotypes in lung cancer. Elife.

[24] Su C., Jiang J., Zhang S., Shi J., Xu K., Shen N., Zhang J., Li L., Zhao L., Zhang J. (2019). Radiomics based on multicontrast MRI can precisely differentiate among glioma subtypes and predict tumour-proliferative behaviour. Eur. Radiol..

[25] Park J.E., Kim H.S., Jo Y., Yoo R.E., Choi S.H., Nam S.J., Kim J.H. (2020). Radiomics prognostication model in glioblastoma using diffusion- and perfusion-weighted MRI. Sci. Rep..

[26] Macyszyn L., Akbari H., Pisapia J.M., Da X., Attiah M., Pigrish V., Bi Y., Pal S., Davuluri R.V., Roccograndi L. (2016). Imaging patterns predict patient survival and molecular subtype in glioblastoma via machine learning techniques. Neuro Oncol..

[27] Aquilanti E., Miller J., Santagata S., Cahill D.P., Brastianos P.K. (2018). Updates in prognostic markers for gliomas. Neuro Oncol..

[28] European Society of R. (2020). ESR Statement on the Validation of Imaging Biomarkers. Insights Imaging.

[29] Radiomics Features. https://pyradiomics.readthedocs.io/en/latest/features.html.

[30] Aerts H.J., Velazquez E.R., Leijenaar R.T., Parmar C., Grossmann P., Carvalho S., Bussink J., Monshouwer R., Haibe-Kains B., Rietveld D. (2014). Decoding tumour phenotype by noninvasive imaging using a quantitative radiomics approach. Nat. Commun..

[31] Gittleman H., Lim D., Kattan M.W., Chakravarti A., Gilbert M.R., Lassman A.B., Lo S.S., Machtay M., Sloan A.E., Sulman E.P. (2017). An independently validated nomogram for individualized estimation of survival among patients with newly diagnosed glioblastoma: NRG Oncology RTOG 0525 and 0825. Neuro Oncol..

[32] Peeken J.C., Goldberg T., Pyka T., Bernhofer M., Wiestler B., Kessel K.A., Tafti P.D., Nusslin F., Braun A.E., Zimmer C. (2019). Combining multimodal imaging and treatment features improves machine learning-based prognostic assessment in patients with glioblastoma multiforme. Cancer Med..

[33] Mazurowski M.A., Desjardins A., Malof J.M. (2013). Imaging descriptors improve the predictive power of survival models for glioblastoma patients. Neuro Oncol..

[34] Nicolasjilwan M., Hu Y., Yan C., Meerzaman D., Holder C.A., Gutman D., Jain R., Colen R., Rubin D.L., Zinn P.O. (2015). Addition of MR imaging features and genetic biomarkers strengthens glioblastoma survival prediction in TCGA patients. J. Neuro Radiol..

[35] Pope W.B., Sayre J., Perlina A., Villablanca J.P., Mischel P.S., Cloughesy T.F. (2005). MR imaging correlates of survival in patients with high-grade gliomas. Ajnr. Am. J. Neuro Radiol..

[36] Schoenegger K., Oberndorfer S., Wuschitz B., Struhal W., Hainfellner J., Prayer D., Heinzl H., Lahrmann H., Marosi C., Grisold W. (2009). Peritumoral edema on MRI at initial diagnosis: An independent prognostic factor for glioblastoma?. Eur. J. Neurol..

[37] Wu C.X., Lin G.S., Lin Z.X., Zhang J.D., Liu S.Y., Zhou C.F. (2015). Peritumoral edema shown by MRI predicts poor clinical outcome in glioblastoma. World J. Surg. Oncol..

[38] Henker C., Kriesen T., Glass A., Schneider B., Piek J. (2017). Volumetric quantification of glioblastoma: Experiences with different measurement techniques and impact on survival. J. Neuro Oncol..

[39] Lemee J.M., Clavreul A., Menei P. (2015). Intratumoral heterogeneity in glioblastoma: Don’t forget the peritumoral brain zone. Neuro Oncol..

[40] Petrecca K., Guiot M.C., Panet-Raymond V., Souhami L. (2013). Failure pattern following complete resection plus radiotherapy and temozolomide is at the resection margin in patients with glioblastoma. J. Neuro Oncol..

[41] Prasanna P., Patel J., Partovi S., Madabhushi A., Tiwari P. (2017). Radiomic features from the peritumoral brain parenchyma on treatment-naive multi-parametric MR imaging predict long versus short-term survival in glioblastoma multiforme: Preliminary findings. Eur. Radiol..

[42] Shi J., Yang S., Wang J., Huang S., Yao Y., Zhang S., Zhu W., Shao J. (2019). Analysis of heterogeneity of peritumoral T2 hyperintensity in patients with pretreatment glioblastoma: Prognostic value of MRI-based radiomics. Eur. J. Radiol..

[43] Leijenaar R.T., Carvalho S., Hoebers F.J., Aerts H.J., van Elmpt W.J., Huang S.H., Chan B., Waldron J.N., O′Sullivan B., Lambin P. (2015). External validation of a prognostic CT-based radiomic signature in oropharyngeal squamous cell carcinoma. Acta Oncol..

[44] Chen X., Fang M., Dong D., Liu L., Xu X., Wei X., Jiang X., Qin L., Liu Z. (2019). Development and Validation of a MRI-Based Radiomics Prognostic Classifier in Patients with Primary Glioblastoma Multiforme. Acad. Radiol..

[45] Kickingereder P., Neuberger U., Bonekamp D., Piechotta P.L., Gotz M., Wick A., Sill M., Kratz A., Shinohara R.T., Jones D.T.W. (2018). Radiomic subtyping improves disease stratification beyond key molecular, clinical, and standard imaging characteristics in patients with glioblastoma. Neuro Oncol..

[46] Kickingereder P., Burth S., Wick A., Gotz M., Eidel O., Schlemmer H.P., Maier-Hein K.H., Wick W., Bendszus M., Radbruch A. (2016). Radiomic Profiling of Glioblastoma: Identifying an Imaging Predictor of Patient Survival with Improved Performance over Established Clinical and Radiologic Risk Models. Radiology.

[47] Choi Y., Nam Y., Jang J., Shin N.Y., Lee Y.S., Ahn K.J., Kim B.S., Park J.S., Jeon S.S., Hong Y.G. (2020). Radiomics may increase the prognostic value for survival in glioblastoma patients when combined with conventional clinical and genetic prognostic models. Eur. Radiol..

[48] Kickingereder P., Bonekamp D., Nowosielski M., Kratz A., Sill M., Burth S., Wick A., Eidel O., Schlemmer H.P., Radbruch A. (2016). Radiogenomics of Glioblastoma: Machine Learning-based Classification of Molecular Characteristics by Using Multiparametric and Multiregional MR Imaging Features. Radiology.

[49] Gupta A., Young R.J., Shah A.D., Schweitzer A.D., Graber J.J., Shi W., Zhang Z., Huse J., Omuro A.M. (2015). Pretreatment Dynamic Susceptibility Contrast MRI Perfusion in Glioblastoma: Prediction of EGFR Gene Amplification. Clin. Neuro Radiol..

[50] Hu L.S., Ning S., Eschbacher J.M., Baxter L.C., Gaw N., Ranjbar S., Plasencia J., Dueck A.C., Peng S., Smith K.A. (2017). Radiogenomics to characterize regional genetic heterogeneity in glioblastoma. Neuro Oncol..

[51] Han Y., Yan L.F., Wang X.B., Sun Y.Z., Zhang X., Liu Z.C., Nan H.Y., Hu Y.C., Yang Y., Zhang J. (2018). Structural and advanced imaging in predicting MGMT promoter methylation of primary glioblastoma: A region of interest based analysis. BMC Cancer.

[52] Leather T., Jenkinson M.D., Das K., Poptani H. (2017). Magnetic Resonance Spectroscopy for Detection of 2-Hydroxyglutarate as a Biomarker for IDH Mutation in Gliomas. Metabolites.

[53] Lohmann P., Lerche C., Bauer E.K., Steger J., Stoffels G., Blau T., Dunkl V., Kocher M., Viswanathan S., Filss C.P. (2018). Predicting IDH genotype in gliomas using FET PET radiomics. Sci. Rep..

[54] Park J.E., Kim H.S., Kim D., Park S.Y., Kim J.Y., Cho S.J., Kim J.H. (2020). A systematic review reporting quality of radiomics research in neuro-oncology: Toward clinical utility and quality improvement using high-dimensional imaging features. BMC Cancer.

[55] Peeken J.C., Hesse J., Haller B., Kessel K.A., Nusslin F., Combs S.E. (2018). Semantic imaging features predict disease progression and survival in glioblastoma multiforme patients. Strahlenther Onkol..

[56] Bae S., Choi Y.S., Ahn S.S., Chang J.H., Kang S.G., Kim E.H., Kim S.H., Lee S.K. (2018). Radiomic MRI Phenotyping of Glioblastoma: Improving Survival Prediction. Radiology.

[57] Moradmand H., Aghamiri S.M.R., Ghaderi R. (2020). Impact of image preprocessing methods on reproducibility of radiomic features in multimodal magnetic resonance imaging in glioblastoma. J. Appl. Clin. Med. Phys..

[58] Shiri I., Hajianfar G., Sohrabi A., Abdollahi H., Shayesteh S.P., Geramifar P., Zaidi H., Oveisi M., Rahmim A. (2020). Repeatability of Radiomic Features in Magnetic Resonance Imaging of Glioblastoma: Test-Retest and Image Registration Analyses. Med. Phys..

[59] Um H., Tixier F., Bermudez D., Deasy J.O., Young R.J., Veeraraghavan H. (2019). Impact of image preprocessing on the scanner dependence of multi-parametric MRI radiomic features and covariate shift in multi-institutional glioblastoma datasets. Phys. Med. Biol..

[60] Rai R., Holloway L.C., Brink C., Field M., Christiansen R.L., Sun Y., Barton M.B., Liney G.P. (2020). Multicenter evaluation of MRI-based radiomic features: A phantom study. Med. Phys..

[61] Niyazi M., Brada M., Chalmers A.J., Combs S.E., Erridge S.C., Fiorentino A., Grosu A.L., Lagerwaard F.J., Minniti G., Mirimanoff R.O. (2016). ESTRO-ACROP guideline “target delineation of glioblastomas”. Radiother. Oncol..

[62] Pavic M., Bogowicz M., Wurms X., Glatz S., Finazzi T., Riesterer O., Roesch J., Rudofsky L., Friess M., Veit-Haibach P. (2018). Influence of inter-observer delineation variability on radiomics stability in different tumor sites. Acta Oncol..

[63] Chang K., Beers A.L., Bai H.X., Brown J.M., Ly K.I., Li X., Senders J.T., Kavouridis V.K., Boaro A., Su C. (2019). Automatic assessment of glioma burden: A deep learning algorithm for fully automated volumetric and bidimensional measurement. Neuro Oncol..

[64] Rios Velazquez E., Meier R., Dunn W.D., Alexander B., Wiest R., Bauer S., Gutman D.A., Reyes M., Aerts H.J. (2015). Fully automatic GBM segmentation in the TCGA-GBM dataset: Prognosis and correlation with VASARI features. Sci. Rep..

[65] Chaddad A., Sabri S., Niazi T., Abdulkarim B. (2018). Prediction of survival with multi-scale radiomic analysis in glioblastoma patients. Med. Biol. Eng. Comput..

[66] Li Z.C., Bai H., Sun Q., Zhao Y., Lv Y., Zhou J., Liang C., Chen Y., Liang D., Zheng H. (2018). Multiregional radiomics profiling from multiparametric MRI: Identifying an imaging predictor of IDH1 mutation status in glioblastoma. Cancer Med..

[67] Park J.E., Kim H.S., Kim N., Park S.Y., Kim Y.H., Kim J.H. (2020). Spatiotemporal Heterogeneity in Multiparametric Physiologic MRI Are Associated with Patient Outcomes in IDH-wildtype Glioblastoma. Clin. Cancer Res..

[68] Tustison N.J., Avants B.B., Cook P.A., Zheng Y., Egan A., Yushkevich P.A., Gee J.C. (2010). N4ITK: Improved N3 bias correction. IEEE Trans. Med. Imaging.

[69] van der Walt S., Schonberger J.L., Nunez-Iglesias J., Boulogne F., Warner J.D., Yager N., Gouillart E., Yu T. (2014). scikit-image: Image processing in Python. PeerJ.

[70] Kaur H., Rani J. MRI brain image enhancement using Histogram Equalization techniques. Proceedings of the 2016 International Conference on Wireless Communications, Signal Processing and Networking (WiSPNET).

[71] Reinhold J.C., Dewey B.E., Carass A., Prince J.L. (2019). Evaluating the Impact of Intensity Normalization on MR Image Synthesis. Proc. SPIE Int. Soc. Opt. Eng..

[72] van Griethuysen J.J.M., Fedorov A., Parmar C., Hosny A., Aucoin N., Narayan V., Beets-Tan R.G.H., Fillion-Robin J.C., Pieper S., Aerts H. (2017). Computational Radiomics System to Decode the Radiographic Phenotype. Cancer Res..

[73] de Leng W.W., Gadellaa-van Hooijdonk C.G., Barendregt-Smouter F.A., Koudijs M.J., Nijman I., Hinrichs J.W., Cuppen E., van Lieshout S., Loberg R.D., de Jonge M. (2016). Targeted Next Generation Sequencing as a Reliable Diagnostic Assay for the Detection of Somatic Mutations in Tumours Using Minimal DNA Amounts from Formalin Fixed Paraffin Embedded Material. PLoS ONE.

[74] van Riet J., Krol N.M.G., Atmodimedjo P.N., Brosens E., van I.W.F.J., Jansen M., Martens J.W.M., Looijenga L.H., Jenster G., Dubbink H.J. (2018). SNPitty: An Intuitive Web Application for Interactive B-Allele Frequency and Copy Number Visualization of Next-Generation Sequencing Data. J. Mol. Diagn.

[75] Jeuken J.W., Cornelissen S.J., Vriezen M., Dekkers M.M., Errami A., Sijben A., Boots-Sprenger S.H., Wesseling P. (2007). MS-MLPA: An attractive alternative laboratory assay for robust, reliable, and semiquantitative detection of MGMT promoter hypermethylation in gliomas. Lab. Investig..

[76] Mogensen U.B., Ishwaran H., Gerds T.A. (2012). Evaluating Random Forests for Survival Analysis using Prediction Error Curves. J. Stat. Softw..

[77] van Timmeren J.E., Leijenaar R.T.H., van Elmpt W., Reymen B., Oberije C., Monshouwer R., Bussink J., Brink C., Hansen O., Lambin P. (2017). Survival prediction of non-small cell lung cancer patients using radiomics analyses of cone-beam CT images. Radiother. Oncol..

[78] Royston P., Altman D.G. (2013). External validation of a Cox prognostic model: Principles and methods. BMC Med. Res. Methodol..

[79] Welch M.L., McIntosh C., Haibe-Kains B., Milosevic M.F., Wee L., Dekker A., Huang S.H., Purdie T.G., O’Sullivan B., Aerts H. (2019). Vulnerabilities of radiomic signature development: The need for safeguards. Radiother. Oncol..

[80] Sanduleanu S., Woodruff H.C., de Jong E.E.C., van Timmeren J.E., Jochems A., Dubois L., Lambin P. (2018). Tracking tumor biology with radiomics: A systematic review utilizing a radiomics quality score. Radiother. Oncol..

[81] Collins G.S., Reitsma J.B., Altman D.G., Moons K.G. (2015). Transparent Reporting of a multivariable prediction model for Individual Prognosis or Diagnosis (TRIPOD): The TRIPOD statement. Ann. Intern. Med..

