# Supplementary material for: Prognostic and Predictive Value of Integrated Qualitative and Quantitative Magnetic Resonance Imaging Analysis in Glioblastoma
Source: Cancers (Basel). 2021 Feb 10;13(4):722. doi: 10.3390/cancers13040722 (PMC7916478; doi:10.3390/cancers13040722)
Supplement: Supplementary file 1 [file cancers-13-00722-s001.pdf]

cancers

MDPI

Supplementary Materials:
Prognostic and predictive value of integrated qualitative and quantitative magnetic resonance imaging analysis in glioblastoma

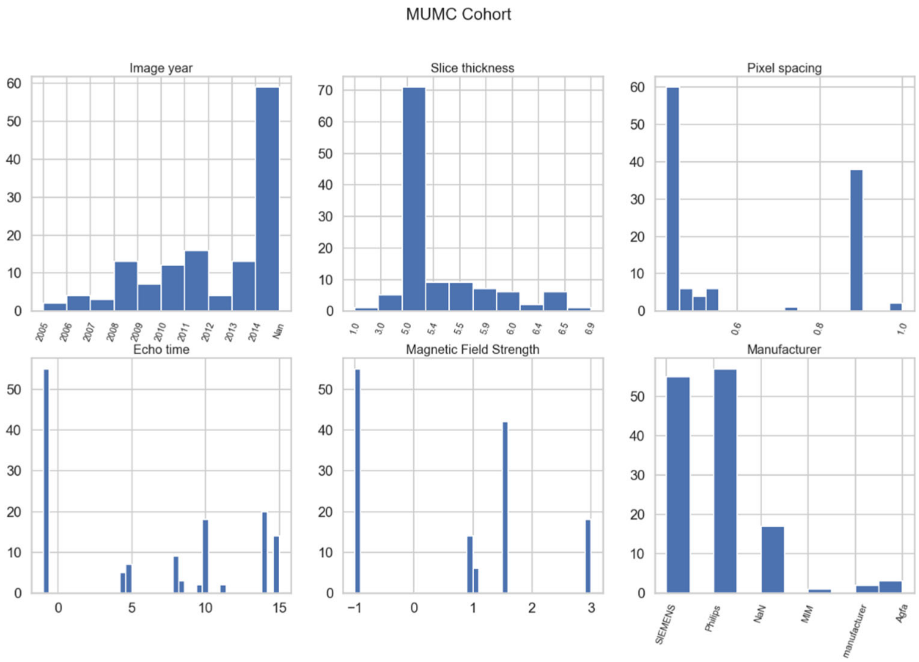

Figure S1. Imaging heterogeneity in Maastricht University Medical Center (MUMC+) cohort. Distribution of values of scanner settings in the cohort. For some images values were lost due to the pseudonymization process.

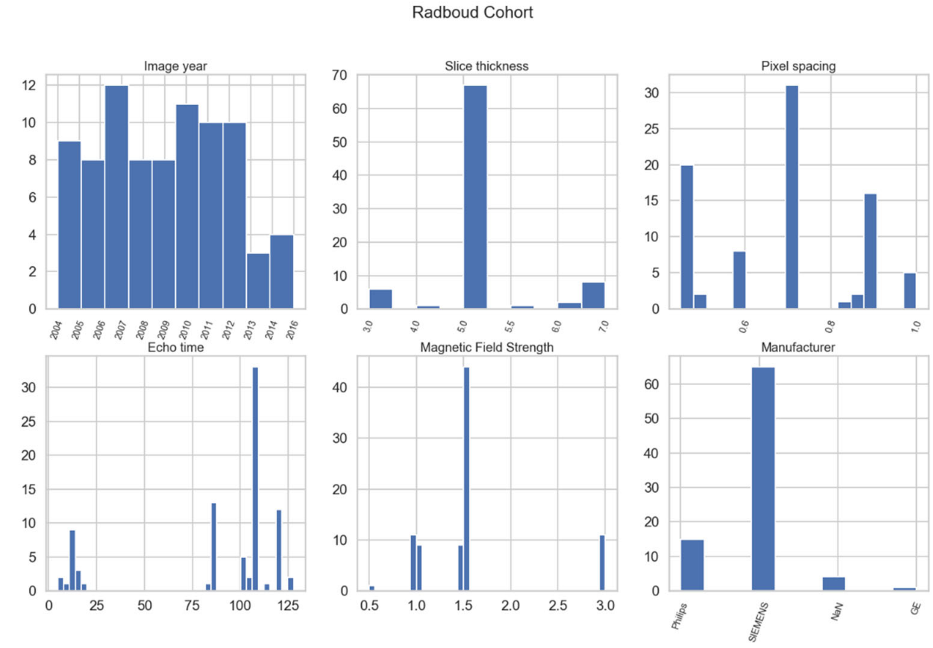

Figure S2. Imaging heterogeneity in Radboudumc cohort. Distribution of values of scanner settings in the cohort.

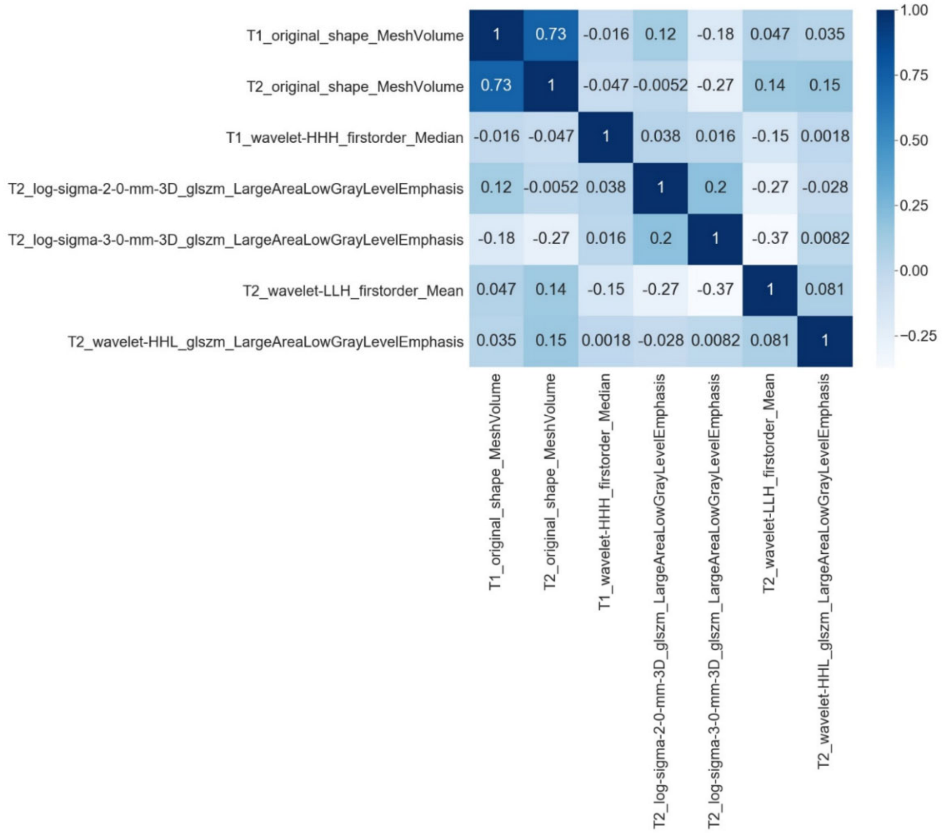

Figure S3. Correlation matrix between radiomics features and tumor volume. This was investigated since previous studies have shown some radiomics features to be surrogate markers for tumor volume and not independent prognostic factors [1]. Correlation was assessed using Spearman's rank correlation.

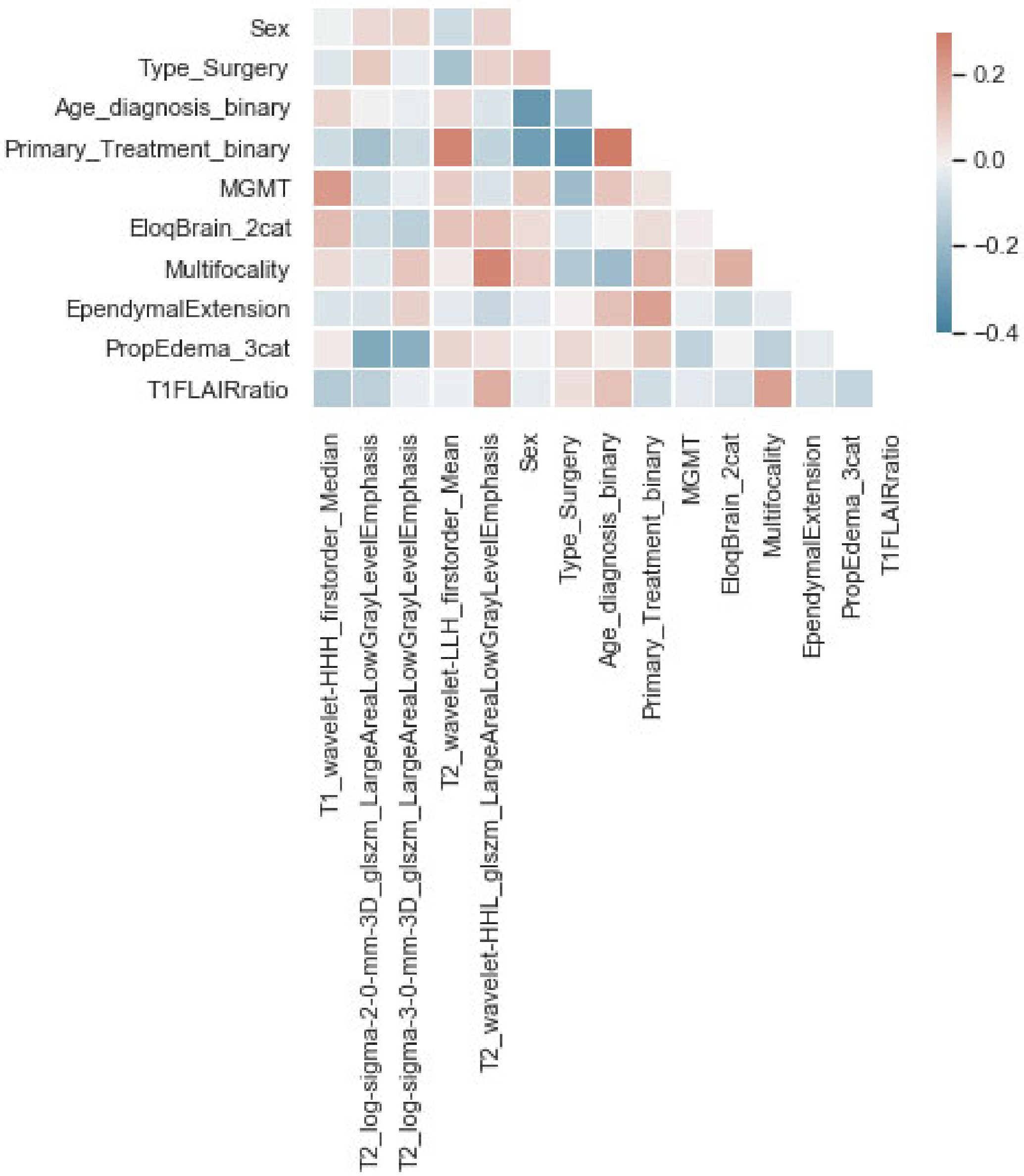

Figure S4. Correlation matrix between VASARI features, clinical features and radiomics features. Correlation was assessed using Point-Biserial Correlation Coefficient.

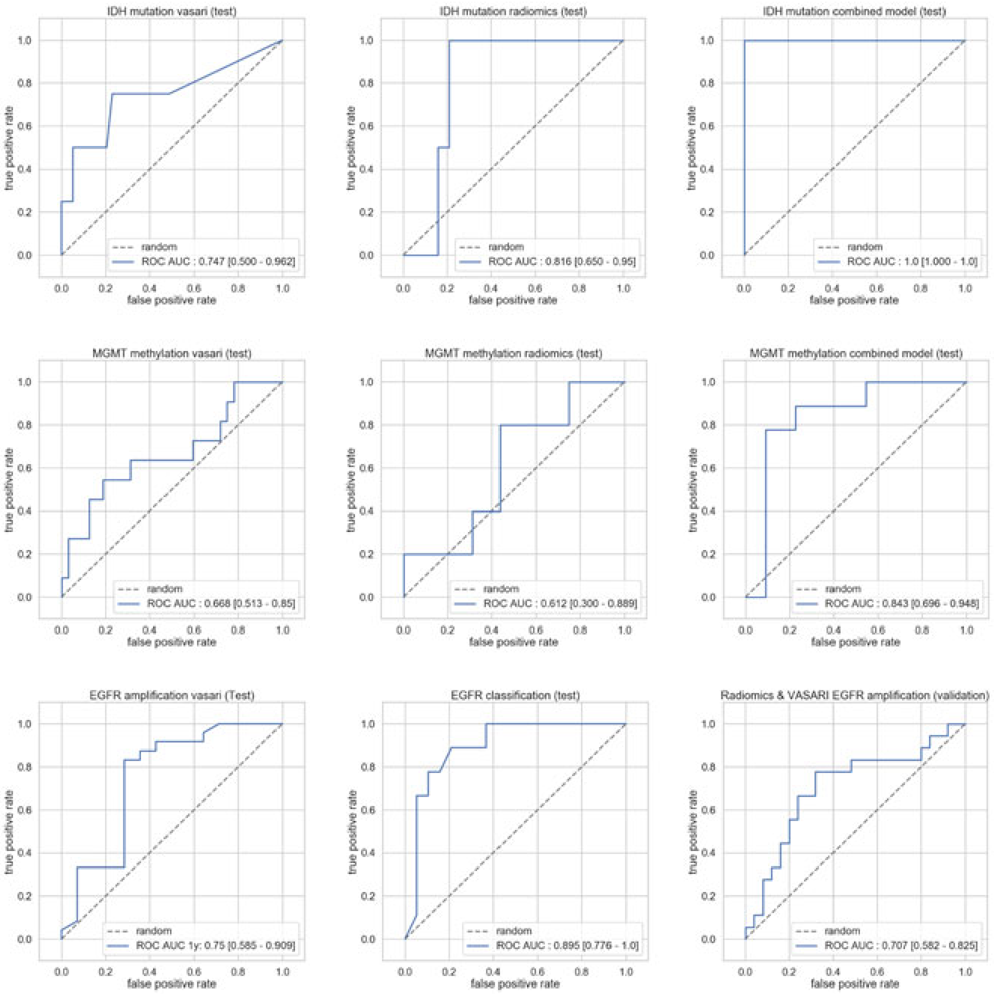

Figure S5. ROC curves for predictive models for isocitrate dehydrogenase (IDH)-mutation, methylguanine methyltransferase (MGMT)-methylation and epidermal growth factor (EGFR) amplification in the test dataset. Performance is shown for each outcome using VASARI features alone (left row), Radiomics features alone (middle row) or VASARI and Radiomics combined (right row). AUC values and 95% confidence intervals are reported.

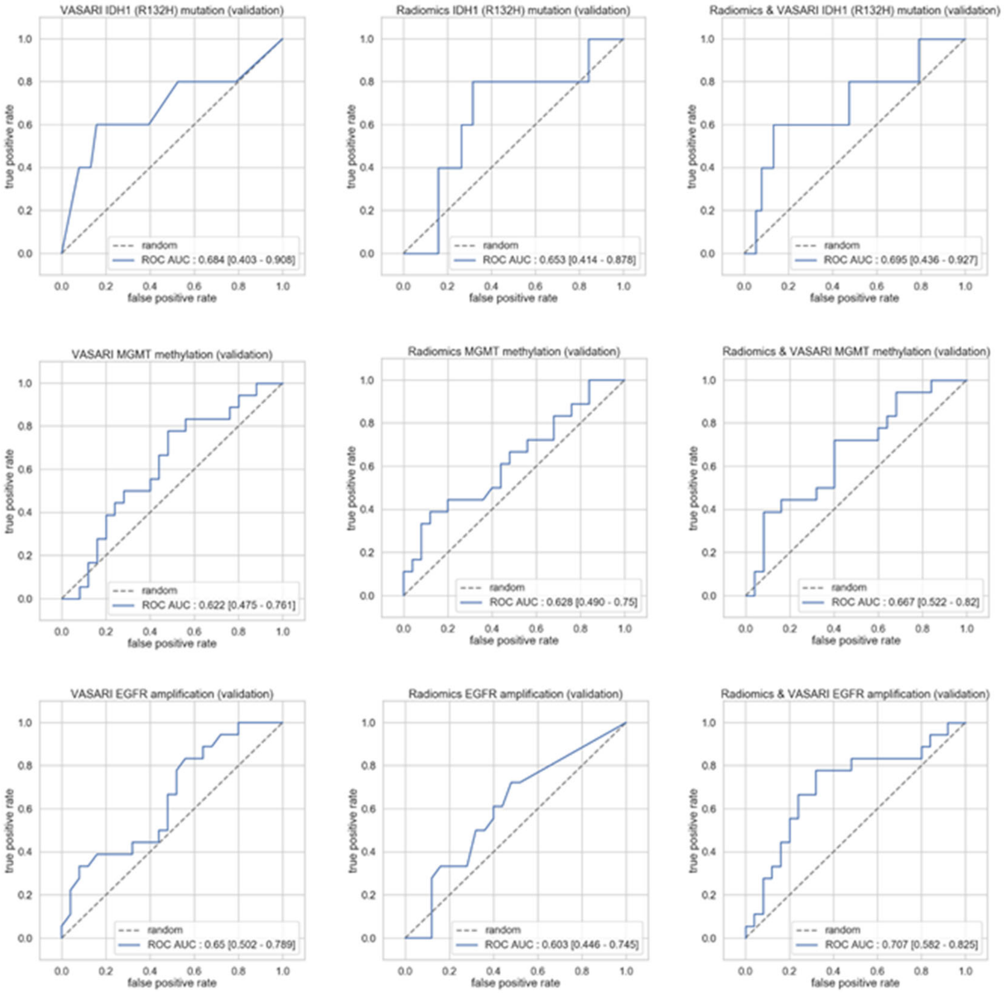

Figure S6. ROC curves for predictive models for isocitrate dehydrogenase (IDH)-mutation, methylguanine methyltransferase (MGMT)-methylation and epidermal growth factor (EGFR) amplification in the validation dataset. Performance is shown for each outcome using VASARI features alone (left row), Radiomics features alone (middle row) or VASARI and Radiomics combined (right row). AUC values and 95% confidence intervals are reported.

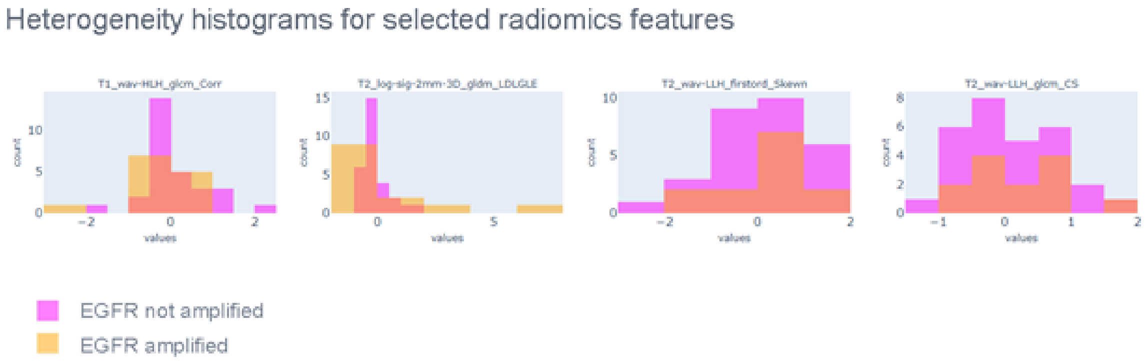

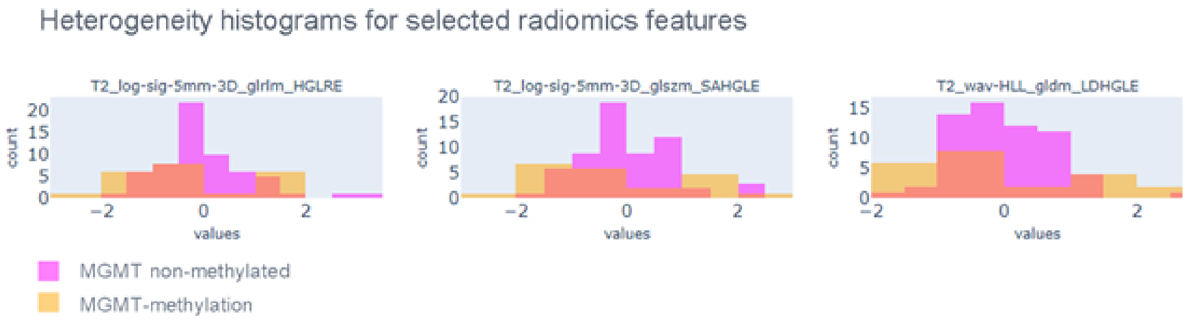

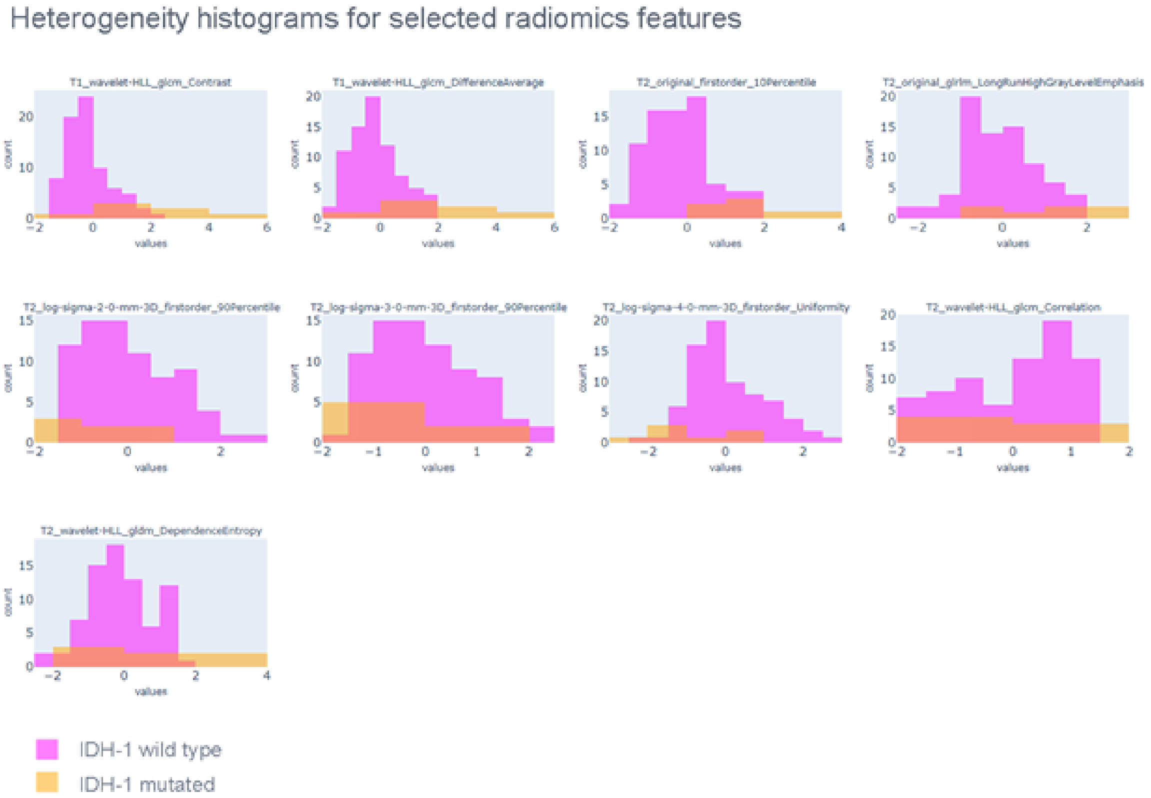

Figure S7. Heterogeneity histograms from the selected radiomics features in predictive models. To visualize the ability of radiomics features of capturing the outcome heterogeneity in a univariate manner the outcome heterogeneity is visualized

through selected radiomics features by plotting the distribution of feature values for each particular feature in the predictive models for epidermal growth factor receptor (EGFR) amplification, methylguanine methyltransferase (MGMT) methylation and isocitrate dehydrogenase (IDH)1mutation status.

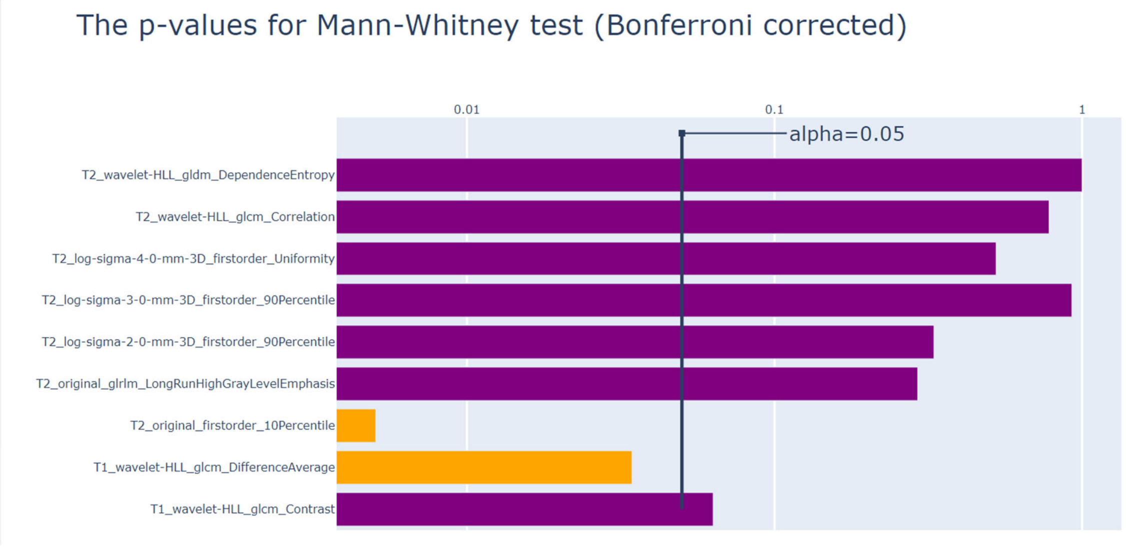

Figure S8. Mann-Whitney test for significance of histogram heterogeneity of radiomics features in predicting isocitrate dehydrogenase (IDH)1 mutation status. Significance is reported for the difference in mean values and after applying Bonferroni correction. No significance was found for histogram heterogeneity of radiomics features for epidermal growth factor receptor (EGFR) amplification or methylguanine methyltransferase (MGMT) methylation (data not shown).

Table S1. Overview of numbers of patients used for the development and validation of each mode presented in this study. Differences between numbers can be explained due to missing MR images needed for radiomics analysis or insufficient quality of images.

Outcome	VASARI[1]	Radiomics[2]	VASARI& Radiomics[3]	Clinical, VASARI&Clinical, VASARI&Radiomics[4]	VASARI& Radiomics& Clinical[5]
Overall survival isocitrate dehydrogenase (IDH)-WT GBM	Train: 129 Validation: 38	Train: 95 Validation: 38	Train: 95 Validation: 38	Train: 95 Validation: 38	Train: 95 Validation: 38
Epidermal growth factor receptor (EGFR) amplification	Train: 64 Test: 28 Validation: 44	Train: 64 Test: 28 Validation: 44	Train: 64 Test: 28 Validation: 44	N.A.	N.A.
Methylguanine methyltransferase (MGMT)-methylation	Train: 74 Test: 30 Validation: 43	Train: 74 Test: 30 Validation: 43	Train: 74 Test: 30 Validation: 43	N.A.	N.A.
IDH1-mutation status	Train: 72 Test: 30 Validation: 43	Train: 72 Test: 30 Validation: 43	Train: 72 Test: 30 Validation: 43	N.A.	N.A.

[1]For all VASARI patients T1+C and T2 and/or FLAIR MR images were available. For 20 patients in the training cohort and 5 patients in the validation cohort no FLAIR image was available and T2 was used instead for features involving FLAIR.
[2]For all Radiomics patients T1+C and T2 MR images were available. Patients for which only FLAIR and not T2 images were available were not included in this group.
[3]For VASARI&Radiomics model all patients in the Radiomics cohort were included.

[4]For Clinical model all patients in the Radiomics cohort were included.
[5]For the Combined model all patients in the Radiomics cohort were included.

Table S2. Univariate Cox-regression analysis of VASARI features for overall survival in isocitrate dehydrogenase (IDH)-wild type glioblastoma population.

**VASARI feature**	**Overall Survival**		
	HR	(95% CI)	p-value
Major axis (mm)	1.072	0.994-1.156	0.071
Major axis (median cut-off) (<6,9 vs. >6,9)	1.505	1.047-2.164	0.027
Major axis (mean cut-off) (<7,00 vs. >7,00)	1.440	1.003-2.068	0.048
Minor axis (mm)	1.044	0.935-1.165	0.442
Minor axis (mean cut-off) (<4,80 vs. >4,80)	1.318	0.919-1.890	0.134
Minor axis (median cut-off) (<4,65 vs. >4,65)	1.472	1.012-2.140	0.043
Tumor location (frontal, temporal, parietal, occipital, insular, basal ganglia, thalamus, brainstem, cerebellum, corpus callosum)			n.s.
Tumor side (right, central/bilateral, left)			n.s.
Involvement of eloquent brain (yes vs. no)	1.359	0.940-1.965	0.103
Enhancement Quality (mild/marked vs. no)	0.852	0.373-1.950	0.705
Cyst (yes vs. no)	1.949	0.946-4.015	0.070
Distribution (focal vs. non-focal)	1.494	0.877-2.547	0.140
T1/FLAIR ratio (non-expansive vs. expansive) NB : for 109 patients FLAIR was available for T1/FLAIR ratio. For the other 20 patients T2 was used instead.	0.745	0.483-1.149	0.183
Thickness of enhancing margin (thick/nodular/solid vs. thin/no enhancing margin).	1.386	0.829-2.317	0.213
Definition of enhancing margin (poorly defined vs. well defined/no enhancing margin)	1.002	0.551-1.822	0.994
Definition of non-enhancing margin (poorly defined vs. well defined/no non-enhancing margin)	1.154	0.772-1.725	0.485
Haemorrhage (yes vs. no)	0.858	0.597-1.234	0.409
Pial invasion (yes vs. no)	0.857	0.596-1.232	0.404
Subependymal extension (yes vs. no)	1.542	1.076-2.208	0.018
Cortical involvement (yes vs. no)	0.741	0.431-1.275	0.279
Deep white matter invasion (yes vs. no)	1.448	0.990-2.118	0.056
Non-contrast enhancing tumor crosses midline (yes vs. no)	1.343	0.829-2.174	0.231
Contrast-enhancing tumor crosses midline (yes vs. no)	1.291	0.763-2.187	0.342
Satellites (yes vs. no)	1.179	0.727-1.912	0.504
Proportion of contrast-enhancing tumor (≤33%, 34-66%, ≥67%)			n.s.
Proportion of non contrast-enhancing tumor (≤33%, 34-66%, ≥67%)			n.s.
Proportion of necrosis (≤33%, 34-66%, ≥67%)			n.s.
Proportion of edema (≤33%, 34-66%, ≥67%)			1 dummy variable <0.2

Table S3. TRIPOD statement assessment for this study. TRIPOD is a checklist recommended in transparent reporting of a multivariable prediction model for individual prognosis or diagnosis.

No.	Y=yes; N=no; R=referenced; NA=not applicable	Development [D]	External validation [V]	Combined Development & External validation [D+V]
**1**	**Identify the study as developing and/or validating a multivariable prediction model, the target population, and the outcome to be predicted.**			**0**
i	The words developing/development, validation/validating, incremental/added value (or synonyms) are reported in the title	N	N	N
ii	The words prediction, risk prediction, prediction model, risk models, prognostic models, prognostic indices, risk scores (or synonyms) are reported in the title	Y	Y	Y
iii	The target population is reported in the title	Y	Y	Y
iv	The outcome to be predicted is reported in the title	Y	Y	Y
**2**	**Provide a summary of objectives, study design, setting, participants, sample size, predictors, outcome, statistical analysis, results, and conclusions.**			**0**
i	The objectives are reported in the abstract	Y	Y	Y
ii	Sources of data are reported in the abstract *E.g. Prospective cohort, registry data, RCT data.*	Y	Y	Y
iii	The setting is reported in the abstract *E.g. Primary care, secondary care, general population, adult care, or paediatric care. The setting should be reported for both the development and validation datasets, if applicable.*	Y	Y	Y
iv	A general definition of the study participants is reported in the abstract *E.g. patients with suspicion of certain disease, patients with a specific disease, or general eligibility criteria.*	Y	Y	Y
v	The overall sample size is reported in the abstract	N	N	N
vi	The number of events (or % outcome together with overall sample size) is reported in the abstract *If a continuous outcome was studied, score Not applicable (NA).*	N	N	N
vii	Predictors included in the final model are reported in the abstract. For validation studies of well-known models, at least the name/acronym of the validated model is reported *Broad descriptions are sufficient, e.g. 'all information from patient history and physical examination'. Check in the main text whether all predictors of the final model are indeed reported in the abstract.*	N	N	N
viii	The outcome is reported in the abstract	N	N	N
ix	Statistical methods are described in the abstract *For model development, at least the type of statistical model should be reported. For validation studies a quote like "model's discrimination and calibration was assessed" is considered adequate. If done, methods of updating should be reported.*	Y	Y	Y

x	Results for model discrimination are reported in the abstract<br>*This should be reported separately for development and validation if a study includes both development and validation.*	Y	Y	Y
xi	Results for model calibration are reported in the abstract<br>*This should be reported separately for development and validation if a study includes both development and validation.*	N	N	N
xii	Conclusions are reported in the abstract<br>*In publications addressing both model development and validation, there is no need for separate conclusions for both; one conclusion is sufficient.*	N	N	N
**3a**	**Explain the medical context (including whether diagnostic or prognostic) and rationale for developing or validating the multivariable prediction model, including references to existing models.**			**1**
i	The background and rationale are presented	Y	Y	Y
ii	Reference to existing models is included (or stated that there are no existing models)	Y	Y	Y
**3b**	**Specify the objectives, including whether the study describes the development or validation of the model or both.**			**1**
i	It is stated whether the study describes development and/or validation and/or incremental (added) value	Y	Y	Y
**4a**	**Describe the study design or source of data (e.g., randomized trial, cohort, or registry data), separately for the development and validation data sets, if applicable.**			**1**
i	The study design/source of data is described<br>*E.g. Prospectively designed, existing cohort, existing RCT, registry/medical records, case control, case series.*<br>*This needs to be explicitly reported; reference to this information in another article alone is insufficient.*	Y	Y	Y
**4b**	**Specify the key study dates, including start of accrual; end of accrual; and, if applicable, end of follow-up.**			**1**
i	The starting date of accrual is reported	Y	Y	Y
ii	The end date of accrual is reported	Y	Y	Y
iii	The length of follow-up <u>and</u> prediction horizon/time frame are reported, if applicable<br>*E.g. "Patients were followed from baseline for 10 years" and "10-year prediction of…"; notably for prognostic studies with long term follow-up.*<br>*If this is not applicable for an article (i.e. diagnostic study or no follow-up), then score Not applicable (NA).*	Y	Y	Y
**5a**	**Specify key elements of the study setting (e.g., primary care, secondary care, general population) including number and location of centres.**			**1**
i	The study setting is reported (e.g. primary care, secondary care, general population)<br>*E.g.: 'surgery for endometrial cancer patients' is considered to be enough information about the study setting.*	Y	Y	Y
ii	The number of centres involved is reported<br>*If the number is not reported explicitly, but can be concluded from the name of the centre/centres, or if clearly a single centre study, score Yes.*	Y	Y	Y

iii	The geographical location (at least country) of centres involved is reported<br>*If no geographical location is specified, but the location can be concluded from the name of the centre(s), score Yes.*	Y	Y	Y
**5b**	**Describe eligibility criteria for participants.**			**1**
i	In-/exclusion criteria are stated<br>*These should explicitly be stated. Reasons for exclusion only described in a patient flow is not sufficient.*	Y	Y	Y
**5c**	**Give details of treatments received, if relevant.**<br>*(i.e. notably for prognostic studies with long term follow-up)*			**1**
i	Details of any treatments received are described<br>*This item is notably for prognostic modelling studies and is about treatment at baseline or during follow-up. The 'if relevant' judgment of treatment requires clinical knowledge and interpretation.*<br>*If you are certain that treatment was not relevant, e.g. in some diagnostic model studies, score Not applicable.*	Y	Y	Y
**6a**	**Clearly define the outcome that is predicted by the prediction model, including how and when assessed.**			**1**
i	The outcome definition is clearly presented<br>*This should be reported separately for development and validation if a publication includes both.*	Y	Y	Y
ii	It is described how outcome was assessed (including all elements of any composite, for example CVD [e.g. MI, HF, stroke]).	Y	Y	Y
iii	It is described when the outcome was assessed (time point(s) since T0)	Y	Y	Y
**6b**	**Report any actions to blind assessment of the outcome to be predicted.**			**1**
i	Actions to blind assessment of outcome to be predicted are reported<br>*If it is clearly a non-issue (e.g. all-cause mortality or an outcome not requiring interpretation), score Yes. In all other instances, an explicit mention is expected.*	Y	Y	Y
**7a**	**Clearly define all predictors used in developing or validating the multivariable prediction model, including how and when they were measured.**			**1**
i	All predictors are reported<br>*For development, "all predictors" refers to all predictors that potentially could have been included in the 'final' model (including those considered in any univariable analyses).*<br>*For validation, "all predictors" means the predictors in the model being evaluated.*	Y	Y	Y
ii	Predictor definitions are clearly presented	Y	Y	Y
iii	It is clearly described how the predictors were measured	Y	Y	Y
iv	It is clearly described when the predictors were measured	Y	Y	Y
**7b**	**Report any actions to blind assessment of predictors for the outcome and other predictors.**			**0**
i	It is clearly described whether predictor assessments were blinded for outcome<br>*For predictors for which it is clearly a non-issue (e.g. automatic blood pressure measurement, age, sex) and for instances where the predictors were*	Y	Y	Y

	clearly assessed before outcome assessment, score Yes. For all other predictors an explicit mention is expected.			
ii	It is clearly described whether predictor assessments were blinded for the other predictors	N	N	N
**8**	**Explain how the study size was arrived at.**			**1**
i	It is explained how the study size was arrived at<br>*Is there any mention of sample size, e.g. whether this was done on statistical grounds or practical/logistical grounds (e.g. an existing study cohort or data set of a RCT was used)?*	Y	Y	Y
**9**	**Describe how missing data were handled (e.g., complete-case analysis, single imputation, multiple imputation) with details of any imputation method.**			**1**
i	The method for handling missing data (predictors and outcome) is mentioned<br>*E.g. Complete case (explicit mention that individuals with missing values have been excluded), single imputation, multiple imputation, mean/median imputation.*<br>*If there is no missing data, there should be an explicit mention that there is no missing data for all predictors and outcome. If so, score Yes.*<br>*If it is unclear whether there is missing data (from e.g. the reported methods or results), score No.*<br>*If it is clear there is missing data, but the method for handling missing data is unclear, score No.*	Y	Y	Y
ii	If missing data were imputed, details of the software used are given<br>*When under 9i explicit mentioning of no missing data, complete case analysis or no imputation applied, score Not applicable.*	NA	NA	NA
iii	If missing data were imputed, a description of which variables were included in the imputation procedure is given<br>*When under 9i explicit mentioning of no missing data, complete case analysis or no imputation applied, score Not applicable.*	NA	NA	NA
iv	If multiple imputation was used, the number of imputations is reported<br>*When under 9i explicit mentioning of no missing data, complete case analysis or no imputation applied, score Not applicable.*	NA	NA	NA
**10a**	**Describe how predictors were handled in the analyses.**			**1**
i	For continuous predictors it is described whether they were modelled as linear, nonlinear (type of transformation specified) or categorized<br>*A general statement is sufficient, no need to describe this for each predictor separately.*<br>*If no continuous predictors were reported, score Not applicable.*	NA	Not applicable	NA
ii	For categorical or categorized predictors, the cut-points were reported<br>*If no categorical or categorized predictors were reported, score Not applicable.*	Y	Not applicable	Y
iii	For categorized predictors the method to choose the cut-points was clearly described<br>*If no categorized predictors, score Not applicable.*	Y	Not applicable	Y
**10b**	**Specify type of model, all model-building procedures (including any predictor selection), and method for internal validation.**			**0**

i	The type of statistical model is reported<br>*E.g. Logistic, Cox, other regression model (e.g. Weibull, ordinal), other statistical modelling (e.g. neural network)*	Y	Not applicable	Y
ii	The approach used for predictor selection <u>before</u> modelling is described<br>*'Before modelling' means before any univariable or multivariable analysis of predictor-outcome associations.*<br>*If no predictor selection before modelling is done, score Not applicable.*<br>*If it is unclear whether predictor selection before modelling is done, score No.*<br>*If it is clear there was predictor selection before modelling but the method was not described, score No.*	Y	Not applicable	Y
iii	The approach used for predictor selection <u>during</u> modelling is described<br>*E.g. Univariable analysis, stepwise selection, bootstrap, Lasso.*<br>*'During modelling' includes both univariable or multivariable analysis of predictor-outcome associations.*<br>*If no predictor selection during modelling is done (so-called full model approach), score Not applicable.*<br>*If it is unclear whether predictor selection during modelling is done, score No.*<br>*If it is clear there was predictor selection during modelling but the method was not described, score No.*	Y	Not applicable	Y
iv	Testing of interaction terms is described<br>*If it is explicitly mentioned that interaction terms were not addressed in the prediction model, score Yes.*<br>*If interaction terms were included in the prediction model, but the testing is not described, score No.*	N	Not applicable	N
v	Testing of the proportionality of hazards in survival models is described<br>*If no proportional hazard model is used, score Not applicable.*	Y	Not applicable	Y
vi	Internal validation is reported<br>*E.g. Bootstrapping, cross validation, split sample.*<br>*If the use of internal validation is clearly a non-issue (e.g. in case of very large data sets), score Yes. For all other situations an explicit mention is expected.*	Y	Not applicable	Y
**10c**	**For validation, describe how the predictions were calculated.**			**1**
i.	It is described how predictions for individuals (in the validation set) were obtained from the model being validated<br>*E.g. Using the original reported model coefficients with or without the intercept, and/or using updated or refitted model coefficients, or using a nomogram, spreadsheet or web calculator.*	Not applicable	Y	Y
**10d**	**Specify all measures used to assess model performance and, if relevant, to compare multiple models.**<br>*These should be described in methods section of the paper (item 16 addresses the reporting of the results for model performance).*			**0**
i	Measures for model discrimination are described<br>*E.g. C-index / area under the ROC curve.*	Y	Y	Y

ii	Measures for model calibration are described<br>*E.g. calibration plot, calibration slope or intercept, calibration table, Hosmer Lemeshow test, O/E ratio.*	N	N	N
iii	Other performance measures are described<br>*E.g. R2, Brier score, predictive values, sensitivity, specificity, AUC difference, decision curve analysis, net reclassification improvement, integrated discrimination improvement, AIC.*	N	N	N
**10e**	**Describe any model updating (e.g., recalibration) arising from the validation, if done.**			**Not applicable**
i	A description of model-updating is given<br>*E.g. Intercept recalibration, regression coefficient recalibration, refitting the whole model, adding a new predictor*<br>*If updating was done, it should be clear which updating method was applied to score Yes.*<br>*If it is not explicitly mentioned that updating was applied in the study, score this item as 'Not applicable'.*	Not applicable	NA	NA
**11**	**Provide details on how risk groups were created, if done.**<br>*If risk groups were not created, score this item as Yes.*			**1**
i	If risk groups were created, risk group boundaries (risk thresholds) are specified<br>*Score this item separately for development and validation if a study includes both development and validation.*<br>*If risk groups were not created, score this item as not applicable.*	Y	Y	Y
**12**	**For validation, identify any differences from the development data in setting, eligibility criteria, outcome and predictors.**			**1**
i	Differences or similarities in definitions with the development study are described<br>*Mentioning of any differences in all four (setting, eligibility criteria, predictors and outcome) is required to score Yes.*<br>*If it is explicitly mentioned that there were no differences in setting, eligibility criteria, predictors and outcomes, score Yes.*	Not applicable	Y	Y
**13a**	**Describe the flow of participants through the study, including the number of participants with and without the outcome and, if applicable, a summary of the follow-up time. A diagram may be helpful.**			**1**
i	The flow of participants is reported	NA	NA	NA
ii	The number of participants with and without the outcome are reported<br>*If outcomes are continuous, score Not applicable.*	Y	Y	Y
iii	A summary of follow-up time is presented<br>*This notably applies to prognosis studies and diagnostic studies with follow-up as diagnostic outcome.*<br>*If this is not applicable for an article (i.e. diagnostic study or no follow-up), then score Not applicable.*	Y	Y	Y
**13b**	**Describe the characteristics of the participants (basic demographics, clinical features, available predictors), including the number of participants with missing data for predictors and outcome.**			**1**
i	Basic demographics are reported	Y	Y	Y

ii	Summary information is provided for all predictors included in the final developed/validated model	Y	Y	Y
iii	The number of participants with missing data for predictors is reported	Y	Y	Y
iv	The number of participants with missing data for the outcome is reported	Y	Y	Y
**13c**	**For validation, show a comparison with the development data of the distribution of important variables (demographics, predictors and outcome).**			**1**
i	Demographic characteristics (at least age and gender) of the validation study participants are reported along with those of the original development study	Not applicable	Y	Y
ii	Distributions of predictors in the model of the validation study participants are reported along with those of the original development study	Not applicable	Y	Y
iii	Outcomes of the validation study participants are reported along with those of the original development study	Not applicable	Y	Y
**14a**	**Specify the number of participants and outcome events in each analysis.**			**1**
i	The number of participants in each analysis (e.g. in the analysis of each model if more than one model is developed) is specified	Y	Not applicable	Y
ii	The number of outcome events in each analysis is specified (e.g. in the analysis of each model if more than one model is developed) *If outcomes are continuous, score Not applicable.*	Y	Not applicable	Y
**14b**	**If done, report the unadjusted association between each candidate predictor and outcome.**			**0**
i	The unadjusted associations between each predictor and outcome are reported *If any univariable analysis is mentioned in the methods but not in the results, score No.* *If nothing on univariable analysis (in methods or results) is reported, score this item as Not applicable.*	N	Not applicable	N
**15a**	**Present the full prediction model to allow predictions for individuals (i.e., all regression coefficients, and model intercept or baseline survival at a given time point).**			**1**
i	The regression coefficient (or a derivative such as hazard ratio, odds ratio, risk ratio) for each predictor in the model is reported	Y	Not applicable	Y
ii	The intercept or the cumulative baseline hazard (or baseline survival) for at least one time point is reported	Y	Not applicable	Y
**15b**	**Explain how to use the prediction model.**			**0**
i	An explanation (e.g. a simplified scoring rule, chart, nomogram of the model, reference to online calculator, or worked example) is provided to explain how to use the model for individualised predictions.	N	Not applicable	N
**16**	**Report performance measures (with confidence intervals) for the prediction model.** *These should be described in results section of the paper (item 10 addresses the reporting of the methods for model performance).*			**0**

i	A discrimination measure is presented<br>*E.g. C-index / area under the ROC curve.*	Y	Y	Y
ii	The confidence interval (or standard error) of the discrimination measure is presented	Y	Y	Y
iii	Measures for model calibration are described<br>*E.g. calibration plot, calibration slope or intercept, calibration table, Hosmer Lemeshow test, O/E ratio.*	N	N	N
iv	Other model performance measures are presented<br>*E.g. R2, Brier score, predictive values, sensitivity, specificity, AUC difference, decision curve analysis, net reclassification improvement, integrated discrimination improvement, AIC.*	N	N	N
**17**	**If done, report the results from any model updating (i.e., model specification, model performance, recalibration).**<br>*If updating was not done, score this TRIPOD item as 'Not applicable'.*			**Not applicable**
0	Model updating was done<br>*If "No", then answer 17i-17v with "Not applicable"*	Not applicable	N	N
i	The updated regression coefficients for each predictor in the model are reported<br>*If model updating was described as 'not needed', score Yes.*	Not applicable	NA	NA
ii	The updated intercept or cumulative baseline hazard or baseline survival (for at least one time point) is reported<br>*If model updating was described as 'not needed', score Yes.*	Not applicable	NA	NA
iii	The discrimination of the updated model is reported	Not applicable	NA	NA
iv	The confidence interval (or standard error) of the discrimination measure of the updated model is reported	Not applicable	NA	NA
v	The calibration of the updated model is reported	Not applicable	NA	NA

**18**	**Discuss any limitations of the study (such as nonrepresentative sample, few events per predictor, missing data).**			**1**
i	Limitations of the study are discussed<br>*Stating any limitation is sufficient.*	Y	Y	Y
**19a**	**For validation, discuss the results with reference to performance in the development data, and any other validation data.**			**1**
i	Comparison of results to reported performance in development studies and/or other validation studies is given	Not applicable	Y	Y
**19b**	**Give an overall interpretation of the results considering objectives, limitations, results from similar studies and other relevant evidence.**			**1**
i	An overall interpretation of the results is given	Y	Y	Y
**20**	**Discuss the potential clinical use of the model and implications for future research.**			**1**
i	The potential clinical use is discussed<br>*E.g. an explicit description of the context in which the prediction model is to be used (e.g. to identify high risk groups to help direct treatment, or to triage patients for referral to subsequent care).*	Y	Y	Y
ii	Implications for future research are discussed<br>*E.g. a description of what the next stage of investigation of the prediction model should be, such as "We suggest further external validation".*	Y	Y	Y

**21**	**Provide information about the availability of supplementary resources, such as study protocol, web calculator, and data sets.**			**1**
i	Information about supplementary resources is provided	Y	Y	Y
**22**	**Give the source of funding and the role of the funders for the present study.**			**1**
i	The source of funding is reported or there is explicit mention that there was no external funding involved	Y	Y	Y
ii	The role of funders is reported or there is explicit mention that there was no external funding	Y	Y	Y
	**Number of applicable TRIPOD items**			**35**
	**Number of TRIPOD items adhered**			**27**
	**OVERALL adherence to TRIPOD**			**77%**

Table S4. Radiomics Quality Score (RQS) assessment for this study. The RQS is a checklist consisting of 16 components to assess the validity of the radiomics workflow and (external) validation of the models.

No.	Radiomics aspects	Maximum points to be scored	Score for this study
1	Image protocol quality - well-documented image protocols (for example, contrast, slice thickness, energy, etc.) and/or usage of public image protocols allow reproducibility/replicability	+ 1 (if protocols are well-documented) + 1 (if public protocol is used)	0
2	Multiple segmentations - possible actions are: segmentation by different physicians/algorithms/software, perturbing segmentations by (random) noise, segmentation at different breathing cycles. Analyse feature robustness to segmentation variabilities	1	0
3	Phantom study on all scanners - detect inter-scanner differences and vendor-dependent features. Analyse feature robustness to these sources of variability	1	0
4	Imaging at multiple time points - collect images of individuals at additional time points. Analyse feature robustness to temporal variabilities (for example, organ movement, organ expansion/shrinkage)	1	0
5	Feature reduction or adjustment for multiple testing - decreases the risk of overfitting. Overfitting is inevitable if the number of features exceeds the number of samples. Consider feature robustness when selecting features	− 3 (if neither measure is implemented) + 3 (if either measure is implemented)	3

6	Multivariable analysis with non radiomics features (for example, EGFR mutation) - is expected to provide a more holistic model. Permits correlating/inferencing between radiomics and non radiomics features	1	1
7	Detect and discuss biological correlates - demonstration of phenotypic differences (possibly associated with underlying gene–protein expression patterns) deepens understanding of radiomics and biology	1	1
8	Cut-off analyses - determine risk groups by either the median, a previously published cut-off or report a continuous risk variable. Reduces the risk of reporting overly optimistic results	1	1
9	Discrimination statistics - report discrimination statistics (for example, C-statistic, ROC curve, AUC) and their statistical significance (for example, p-values, confidence intervals). One can also apply resampling method (for example, bootstrapping, cross-validation)	+ 1 (if a discrimination statistic and its statistical significance are reported) + 1 (if a resampling method technique is also applied)	2
10	Calibration statistics - report calibration statistics (for example, Calibration-in-the-large/slope, calibration plots) and their statistical significance (for example, *P*-values, confidence intervals). One can also apply resampling method (for example, bootstrapping, cross-validation)	+ 1 (if a calibration statistic and its statistical significance are reported) + 1 (if a resampling method technique is also applied)	1
11	Prospective study registered in a trial database - provides the highest level of evidence supporting the clinical validity and usefulness of the radiomics biomarker	+ 7 (for prospective validation of a radiomics signature in an appropriate trial)	0
12	Validation - the validation is performed without retraining and without adaptation of the cut-off value, provides crucial information with regard to credible clinical performance	- 5 (if validation is missing) + 2 (if validation is based on a dataset from the same institute) + 3 (if validation is based on a dataset from another institute) + 4 (if validation is based on two datasets from two distinct institutes) + 4 (if the study validates a previously published signature) + 5 (if validation is based on three or more datasets from distinct institutes)	3

13	Comparison to 'gold standard' - assess the extent to which the model agrees with/is superior to the current 'gold standard' method (for example, TNM-staging for survival prediction). This comparison shows the added value of radiomics	2	2
14	Potential clinical utility - report on the current and potential application of the model in a clinical setting (for example, decision curve analysis).	2	2
15	Cost-effectiveness analysis - report on the cost-effectiveness of the clinical application (for example, QALYs generated)	1	0
16	Open science and data - make code and data publicly available. Open science facilitates knowledge transfer and reproducibility of the study	+ 1 (if scans are open source) + 1 (if region of interest segmentations are open source) + 1 (if code is open source) + 1 (if radiomics features are calculated on a set of representative ROIs and the calculated features and representative ROIs are open source)	1
	Total score:	36	17

Table S5. Definitions of VASARI features as used in this study. Adapted from *Wangaryattawanich et al [2].*

VASARI feature	Definition
Major axis (mm)	The longest diameter of the tumor which is based upon measurement of the FLAIR (or T2) abnormality on a single axial image that demonstrates the largest cross-sectional area.
Major axis (median cut-off) (<6,9 vs. >6,9)	Binary classification of major axis above or below median value as determined in the training cohort.
Major axis (mean cut-off) (<7,00 vs. >7,00)	Binary classification of major axis above or below mean value as determined in the training cohort.
Minor axis (mm)	The diameter of the FLAIR (or T2) abnormality which is perpendicular to the longest diameter. The measurement is performed on a single asxial image that demonstrates the largest cross-sectional area.
Minor axis (mean cut-off) (<4,80 vs. >4,80)	Binary classification of minor axis above or below median value as determined in the training cohort.
Minor axis (median cut-off) (<4,65 vs. >4,65)	Binary classification of minor axis above or below mean value as determined in the training cohort.
Tumor location (frontal, temporal, parietal, occipital, insular, basal ganglia, thalamus, brainstem, cerebellum, corpus callosum)	Location of (largest portion of) the tumor, (including both contrast-enhancing (CET) or non-contrast-enhancing tumor (nCET).
Tumor side (right, central/bilateral, left)	Side of lesion epicentre irrespective of whether lesion crosses into the contralateral hemisphere.

Involvement of eloquent brain (yes vs. no)	Presence of tumor involvement in the eloquent cortex (speech motor, speech receptive, motor or vision).
Enhancement Quality (mild/marked vs. no)	Qualitative degree of contrast enhancement (significantly higher signal on postcontrast T1W images compared with precontrast T1W images).
Cyst (yes vs. no)	Well-defined, rouded regions of very bright T2W signal and low T1W signal (matching CSF signal) with tin, regular, smoot, non-enhancing or regularly enhancing walls, possibly with thin, regular, internal septations.
Distribution (focal vs. non-focal)	Non-focal tumors include tumors of which at least one region of tumor (either CET or nCET) is not contiguous with the dominant lesion and outside the region of signal abnormality surrounding the dominant mass.
T1/FLAIR ratio (non-expansive vs. expansive)	Gross composition in the overall lesion size between precontrast T1 and FLAIR (or T2) in the same plan. Expansive (T1=FLAIR) or non-expansive (T1<FLAIR).
Thickness of enhancing margin (thick/nodular/solid vs. thin/no enhancing margin).	The thickness of the enhancing margin of the tumor. Not applicable if there is no contrast enhancement. Thick is considered ≥3mm, thin ≤3mm.
Definition of enhancing margin (poorly defined vs. well defined/no enhancing margin)	The definition of the outside enhancing margin of the tumor. Not applicable if there is no contrast enhancement.
Definition of non-enhancing margin (poorly defined vs. well defined/no non-enhancing margin)	The definition of the outside margin of the non-enhancing margin of the tumor.
Haemorrhage (yes vs. no)	Intrinsic haemorrhage anywhere within the tumor matrix (any foci of low signal on T2WI or high signal on T1WI).
Pial invasion (yes vs. no)	Enhancement of the overlying pia in continuity with enhancing or non-enhancing margin.
Subpendymal extension (yes vs. no)	Invasion of any adjacent ependymal surface in continuity with enhancing or non-enhancing tumor.
Cortical involvement (yes vs. no)	Non-enhancing or enhancing tumor extending to the cortical mantle or cortex.
Deep white matter invasion (yes vs. no)	Enhancing or non-enhancing tumor extending into the internal capsule, corpus callosum or brainstem.
Non-contrast enhancing tumor crosses midline (yes vs. no)	nCET crosses into the contralateral hemisphere through white matter commissures.
Contrast-enhancing tumor crosses midline (yes vs. no)	Enhancing tissue crosses into contralateral hemisphere through with matter commissures.
Satellites (yes vs. no)	An area of enhancement within the region of signal abnormality surrounding the dominant lesion but not continuous with the major enhancing tumor mass.
Proportion of contrast-enhancing tumor (≤33%, 34-66%, ≥67%)	Visually estimated proportion of enhancing component of the entire tumor.
Proportion of non contrast-enhancing tumor (≤33%, 34-66%, ≥67%)	Visually estimated proportion of non-enhancing component of the entire tumor.
Proportion of necrosis (≤33%, 34-66%, ≥67%)	Visually estimated proportion of necrosis to the entire tumor (non-enhancing region with high signal on T2W images and low on T1W images).
Proportion of edema (≤33%, 34-66%, ≥67%)	Visually estimated proportion of edema relative to the entire tumor mass (CET+nCET+necrosis).

Table 6. Tuned hyperparameters used for development of predictive models for isocitrate dehydrogenase (IDH)-mutation, methylguanine methyltransferase (MGMT)-methylation and epidermal growth factor receptor (EGFR) amplification.

Model	Hyper-parameter values
Logistic regression (Scikit-learn version 0.21.3)	number of features: [2:15]<br>Penalty: [L1,L2]
Random Forest (Scikit-learn version 0.21.3)	number of features: [2:15]<br>max depth: [2:7]<br>number of estimators: [50:300]
XGBoost (xgboost version 0.90)	number of features: [2:15]<br>max depth: [2:7]<br>number of estimators: [50:300],<br>learning rate: [0.1, 0.15, 0.2, 0.25, 0.3, 0.01, 0.03, 0.09, 0.001, 0.003]

References

1. Welch, M.L.; McIntosh, C.; Haibe-Kains, B.; Milosevic, M.F.; Wee, L.; Dekker, A.; Huang, S.H.; Purdie, T.G.; O'Sullivan, B.; Aerts, H., et al. Vulnerabilities of radiomic signature development: The need for safeguards. *Radiother Oncol* **2019**, *130*, 2-9, doi:10.1016/j.radonc.2018.10.027.
2. Wangaryattawanich, P.; Hatami, M.; Wang, J.; Thomas, G.; Flanders, A.; Kirby, J.; Wintermark, M.; Huang, E.S.; Bakhtiari, A.S.; Luedi, M.M., et al. Multicenter imaging outcomes study of The Cancer Genome Atlas glioblastoma patient cohort: imaging predictors of overall and progression-free survival. *Neuro Oncol* **2015**, *17*, 1525-1537, doi:10.1093/neuonc/nov117.
